# Assessing the contribution of rare variants to congenital heart disease through a large-scale case-control exome study

**DOI:** 10.1038/s41525-026-00582-z

**Published:** 2026-06-02

**Authors:** Enrique Audain, Anna Wilsdon, Gregor Dombrowsky, Alejandro Sifrim, Jeroen Breckpot, Yasset Perez-Riverol, Siobhan Loughna, Allan Daly, Pavlos Antoniou, Philipp Hofmann, Amilcar Perez-Riverol, Anne-Karin Kahlert, Ulrike Bauer, Thomas Pickardt, Sabine Klaassen, Felix Berger, Ingo Daehnert, Sven Dittrich, Brigitte Stiller, Hashim Abdul-Khaliq, Frances Bu’lock, Anselm Uebing, Hans-Heiner Kramer, Vivek Iyer, Lars Allan Larsen, J. David Brook, Marc-Phillip Hitz

**Affiliations:** 1 Department of Congenital Heart Disease and Pediatric Cardiology, University Hospital of Schleswig-Holstein, Kiel, Germany; 2 Institute for Medical Genetics, Oldenburg, Germany; 3 School of Life Sciences, University of Nottingham, University Park, Nottingham, UK; 4 Department of Human Genetics, KU Leuven, University of Leuven, Leuven, Belgium; 5 European Bioinformatics Institute (EBI), Wellcome Trust Genome Campus, Cambridge, UK; 6 Wellcome Sanger Institute, Wellcome Genome Campus, Cambridge, UK; 7 Competence Network for Congenital Heart Defects, Berlin, Germany; 8 Experimental and Clinical Research Center (ECRC), Charité - Universitätsmedizin Berlin and Max Delbrück Center, Berlin, Germany; 9 Deparment of Congenital Heart Disease-Pediatric Cardiology, Deutsches Herzzentrum der Charité, Berlin, Germany; 10 Department of Pediatric Cardiology and Congenital Heart Disease, Heart Center, University of Leipzig, Leipzig, Germany; 11 Department of Pediatric Cardiology, University Hospital Erlangen, Friedrich-Alexander-University Erlangen-Nürnberg (FAU), Erlangen, Germany; 12 Department of Congenital Heart Disease and Pediatric Cardiology, University Heart Center Freiburg—Bad Krozingen, Freiburg, Germany; 13 Department of pediatric Cardiology, Saarland University Hospital, Homburg, Germany; 14 Congenital and Paediatric Cardiology, East Midlands Congenital Heart Centre, Glenfield Hospital, University of Leicester, Leicester, UK; 15 German Centre for Cardiovascular Research (DZHK), Partner Site Kiel, Kiel, Germany; 16 Department of Cellular and Molecular Medicine, University of Copenhagen, Copenhagen, Denmark

**Keywords:** Gene expression, Genetic interaction, Next-generation sequencing, Congenital heart defects, Genetic association study

## Abstract

Several studies have demonstrated the value of large-scale human exome and genome data analysis to maximise gene discovery in rare diseases. Using this approach, we have analysed the exomes of 4747 cases and 52,881 controls to identify genes which confer a substantial risk of congenital heart disease (CHD). We identified both rare loss-of-function and missense coding variants in 14 genes, which reached genome-wide significance at *FDR* 5%. Ten genes have been associated with CHD, whereas four genes (*PBX1, KAT6B, SHOX2, HCAR1*) have not been reported as genome-wide significant so far. We highlight distinct genetic contributions to syndromic and non-syndromic CHD by independently analysing probands from these two groups. In addition, by integrative analysis of exome data with single-cell transcriptomics data from human embryonic hearts, we identified cardiac-specific cells, such as neural crest cells and endothelial cells, as well as putative biological processes underlying the pathogenesis of CHD. In summary, our findings strengthen the association of known CHD genes and have identified additional novel disease genes contributing to the aetiology of CHD.

## Introduction

Congenital Heart Disease (CHD) is a global health challenge, affecting ~1–2% of live births worldwide^1^. However, despite advances in our understanding of the underlying disease aetiology in recent years, a significant proportion of CHD cases remains unexplained, suggesting that genetic mechanisms and other risk factors remain poorly understood^2,3^. Recent advances in exome and genome sequencing technologies have opened up new avenues of study and have resulted in novel insights into the genetic and epigenetic mechanisms underlying rare diseases, such as CHD^4,5^.

Previous studies have defined the association of inherited and de novo variations as a cause of CHD^6,7^. In addition, these studies have highlighted the differences between the genetic architecture of syndromic (with extracardiac malformations and/or neurodevelopmental delay) and non-syndromic (isolated) CHD^6,7^. Continuing collaboration between the scientific community and healthcare teams has driven efforts to integrate and analyse larger cohorts of patients, and has demonstrated the potential of this approach to uncover novel variants and genes associated with CHD^6–8^.

Here, we present a whole-exome sequencing analysis of 4747 CHD cases and 52,881 controls. This is one of the largest cohorts of non-syndromic CHD cases (*n* = 2929) studied so far, meaning that we are in an advantageous position to refine our understanding of the genetic mechanisms underlying non-syndromic CHD specifically. This is especially important given that the vast majority of individuals with CHD (~75%) have non-syndromic CHD^6,7^.

We used the case-control cohort to investigate monogenic factors contributing to CHD. We integrated data obtained in the case-control study with single-cell transcriptome data obtained from human embryonic hearts^9^. This complementary analysis identified biological processes enriched for genes differentially expressed in cardiac-specific cells, found also significant in our case-control analysis. Importantly, the data suggest a difference in cardiac developmental mechanisms between syndromic and non-syndromic CHD.

Taken together, we have identified ten genome-wide significant (Bonferroni adjusted *P* < 0.05) genes and an additional four genes at FDR 5%, which are associated with CHD.

## Results

### Cohort description and analysis workflow

We combined and analysed the exomes of 4747 CHD cases (aCHD, refers to all CHD cases) and 52,881 controls. CHD cases were further classified into syndromic CHD (sCHD, individuals with extracardiac malformations or neurodevelopmental disability, *n* = 1818) and non-syndromic CHD (nsCHD, individuals with isolated CHD, *n* = 2929). Detailed information about patient phenotype, ancestry, age and gender is summarised in Supplementary Data 1. An overview of cardiac sub-phenotypes distribution across the case cohort is represented in Supplementary Fig. 1. All samples and genetic variants were subjected to a sequence of quality control steps to obtain a final cohort of unrelated and matched-ancestry individuals, as well as a set of high-confidence variants for downstream analysis (see Methods and Supplementary Information).

**Fig. 1:**
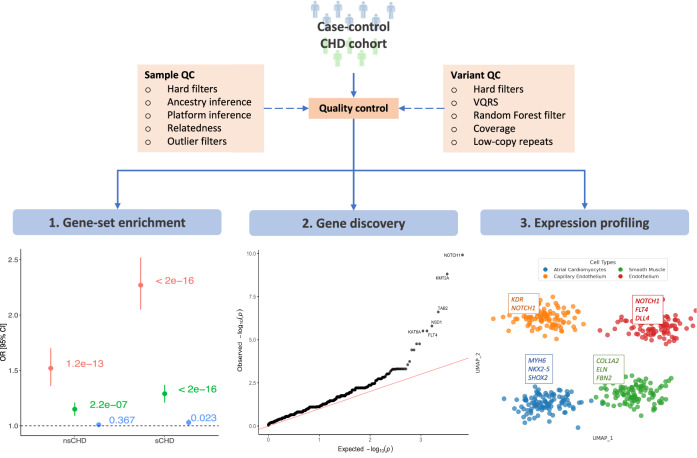
Analysis workflow for disease gene discovery. Quality control processes were conducted at the sample and variant levels. **1** Gene-set enrichment analysis was performed on the gene intolerance to missense and loss-of-function spectrum. **2** Gene-based case-control burden testing (Fisher’s Exact test) was performed for high-confidence loss-of-function (hcLOF) and missense constrained variants (missC) independently. The per gene minimal *p*-value (*P*) from both analyses was set as the study-wide *p*-value, corrected for multiple testing using the Bonferroni and B-H methods. **3** Expression profiling of significant CHD genes differentially expressed on cardiac specific cell clusters.

We evaluated the distribution of high-confidence loss-of-function (hcLOF) and missense constrained variants (missC) across a spectrum of LOF and missense constrained genes (Methods). In addition, we performed gene-based burden testing to identify genes conferring a high risk of CHD, as well as the expression pattern at single-cell resolution. Figure 1 simplifies the workflow followed in this study to discover novel associations with CHD.

### Distinct pattern of loss-of-function constrained genes identified between sCHD and nsCHD

Previous studies have suggested a greater contribution of loss-of-function (LOF) variants to sCHD, compared to non-syndromic forms^6,7^. To determinate if this holds true in this present cohort, we evaluated the burden of rare variants in the sCHD and nsCHD cohort, compared with controls across the per gene spectrum of loss-of-function intolerance. Following the approach proposed by the gnomAD consortium^10^, we divided 19,923 protein-coding genes into ten bins (~1900 genes per bin) based on its observed/expected LOF ratio upper fraction (termed LOEUF) and applied a logistic regression model (see Methods) to each bin (i.e., gene-set). This allowed us to assess enrichment across three different functional categories of variants (hcLOF, missC and synonymous), stratified by CHD probands (aCHD, sCHD and nsCHD).

**Fig. 2:**
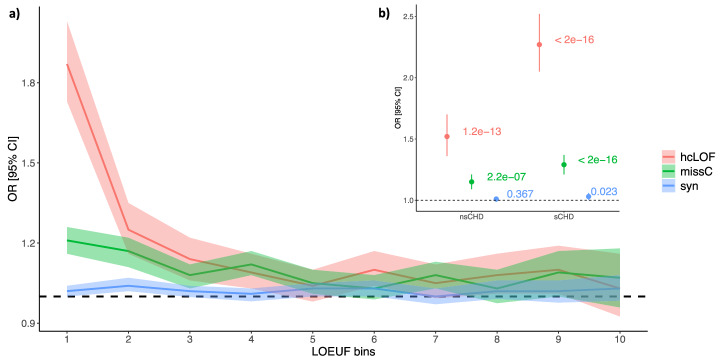
Enrichment analysis across the LOF constraint gene spectrum. Protein-coding genes were binned based on the LOEUF metric as proposed by gnomAD. Every bin contains ~1900 genes. Top bins (1, 2) contain genes with the highest intolerance to loss-of-function. **a** Enrichment analysis comparing aCHD vs. controls. **b** Enrichment analysis stratified by syndromic status (sCHD and nsCHD) vs. controls in the top constraint LOF bin (1). The x-axis indicates the constraint bins; the y-axis shows the Odds Ratios (OR) and the 95% confidence interval.

The highest enrichment was observed in the most LOF-constrained genes (bin 1) for hcLOF variants (Fig. 2 and Supplementary Data 2). These variants provided a major contribution to sCHD cases (*OR* = 2.27, *P* < 2 × 10^−16^), and much less so for nsCHD (*OR* = 1.52, *P* = 1.2 × 10^−13^). A moderate enrichment was observed for missC variations, suggesting that this class of variants could have a similar (although smaller) functional impact compared to hcLOF variants. The observed effect may be explained by the defined set of constrained missense variants likely operating through a haploinsufficient mechanism. Although reduced in magnitude, this same pattern was also observed in the set of genes in the second LOEUF constraint bin, whereas no enrichment was observed towards less LOEUF-constrained bins (Fig. 2). Synonymous variants showed only small and inconsistent deviations from an odds ratio of 1 across LOEUF bins, without a systematic constraint-dependent enrichment, supporting their use as a negative control set (Fig. 2 and Supplementary Data 2).

When the same analysis was performed across the missense constraint spectrum, assessed by the observed/expected missense ratio upper fraction gene-based metric (termed MOEUF), a similar pattern as described above (higher enrichment in the most missense-constrained genes) was observed (Supplementary Fig. 2).

These results demonstrate a larger effect of hcLOF compared to missC variants across the LOEUF and MOEUF spectrum, with the major contribution observed in sCHD, compared with nsCHD. Nevertheless, the results suggest that both hcLOF and missC variants are important genetic components contributing to CHD development.

### Gene-based enrichment analysis

To identify genes that confer a significant risk of CHD, we performed a case-control burden analysis by combining rare variants (MAF < 0.001) at the gene level. It has been demonstrated that following a method of collapsing variants within specific genomic regions (e.g., genes) increases the power to discover new associations at low allele frequencies^11^. Following this principle, we conducted a Fisher’s exact test to identify genes with a significant burden of non-synonymous variants in CHD cases compared to controls, and evaluated them independently for sCHD and nsCHD.

As with earlier comparable case-control exome studies^12–14^, the burden test was performed separately for hcLOF (*P*_lof_) and missC (*P*_miss_), and the minimal *p*-value observed per gene between these two variant categories was selected as the study-wide *p*-value (*P*). hcLOF variants were defined using the LOFTEE tool^10^, whereas missense variants were defined based on different missense deleteriousness prediction scores (see Methods and Supplementary Fig. 3). Ten genes were identified with significant *P*, after correcting for multiple testing using the Bonferroni method (Table 1 and Supplementary Data 3). Eight genes were associated with sCHD (*KMT2A*, *SMAD4*, *PTPN11, TAB2, NSD1, BCOR, KAT6A, PBX1)* and two were identified through the nsCHD (*FLT4* and *NOTCH1*) analysis. In addition, four genes showed significant associations with CHD at *FDR* 5% (*CTCF, KAT6B, SHOX2, HCAR1*). The evaluation of the set of synonymous variants showed a similar distribution of expected vs. observed *p*-values, suggesting negligible genomic inflation of the test statistic (Supplementary Fig. 4).

**Table 1:** Top 21 genes in the case-control burden analysis using the Fisher Exact test stratified by syndromic status (sCHD and nsCHD). A total of 16,351 genes were tested per variant type (hcLOF and missC)

Genes	Analysis	CSQ	sCHD	nsCHD	Controls	*P*	*P* (FDR)	*P* (Bonferroni)	Known CHD
*KMT2A*	sCHD	hcLOF	8	0	0	9.76E-13	3.19E-08	3.19E-08	yes
*SMAD4*	sCHD	missC	11	3	16	2.47E-10	4.04E-06	8.09E-06	yes
*NOTCH1*	nsCHD	hcLOF	2	7	0	8.48E-10	2.77E-05	2.77E-05	yes
*PTPN11*	sCHD	missC	11	5	25	8.78E-09	9.57E-05	2.87E-04	yes
*TAB2*	sCHD	hcLOF	5	1	0	3.13E-08	2.56E-04	1.02E-03	yes
*NSD1*	sCHD	hcLOF	5	1	1	1.83E-07	1.20E-03	5.98E-03	yes
*BCOR*	sCHD	hcLOF	4	0	0	9.93E-07	4.06E-03	3.25E-02	yes
*KAT6A*	sCHD	hcLOF	4	1	0	9.93E-07	4.06E-03	3.25E-02	yes
*PBX1*	sCHD	missC	6	3	6	7.73E-07	4.06E-03	2.53E-02	no
*FLT4*	nsCHD	hcLOF	0	5	0	3.32E-07	5.43E-03	1.09E-02	yes
*CTCF*	sCHD	missC	4	1	1	4.84E-06	1.58E-02	1.58E-01	yes
*KAT6B*	sCHD	hcLOF	4	1	1	4.84E-06	1.58E-02	1.58E-01	no
*SHOX2*	nsCHD	missC	1	10	21	1.81E-06	1.98E-02	5.93E-02	no
*HCAR1*	nsCHD	missC	2	9	18	4.40E-06	3.60E-02	1.44E-01	no
*ADNP*	sCHD	hcLOF	3	0	0	3.15E-05	6.44E-02	1.00E + 00	yes
*CHD7*	sCHD	hcLOF	3	0	0	3.15E-05	6.44E-02	1.00E + 00	yes
*EP300*	sCHD	hcLOF	3	1	0	3.15E-05	6.44E-02	1.00E + 00	yes
*KMT2D*	sCHD	hcLOF	3	0	0	3.15E-05	6.44E-02	1.00E + 00	yes
*KRT25*	sCHD	missC	8	3	31	2.51E-05	6.44E-02	8.19E-01	no
*QRICH1*	sCHD	hcLOF	3	0	0	3.15E-05	6.44E-02	1.00E + 00	no
*SLC38A9*	nsCHD	missC	0	6	6	1.19E-05	7.78E-02	3.89E-01	no

Analysis: sCHD or nsCHD vs. controls. CSQ: denotes the consequence group with the minimal *p*-value (*P*). sCHD: number of syndromic cases (heterozygous). nsCHD: number of non-syndromic cases (heterozygous). Controls: number of controls (heterozygous). *P*: the minimal observed *p-*value per gene between *P*_lof_ and *P*_miss_. P (FDR): Adjusted minimal *p*-value (*P*) using the B-H method with *n* = 2 × 16,351. *P* (Bonferroni): Adjusted minimal *p*-value (*P*) using the Bonferroni method with *n* = 2 × 16,351. In bold are highlighted the ten genes with Bonferroni adjusted *P* < 0.05. Known CHD: Indicates whether the gene has a well-established association with CHD (“yes”) or has not been reported as genome-wide significant thus far (“no”). Supplementary Data 3 contains the results for all protein-coding genes tested. Supplementary Data 6 contains individual and variant-level information for rare protein-altering variants in prioritised CHD genes.

**Fig. 3:**
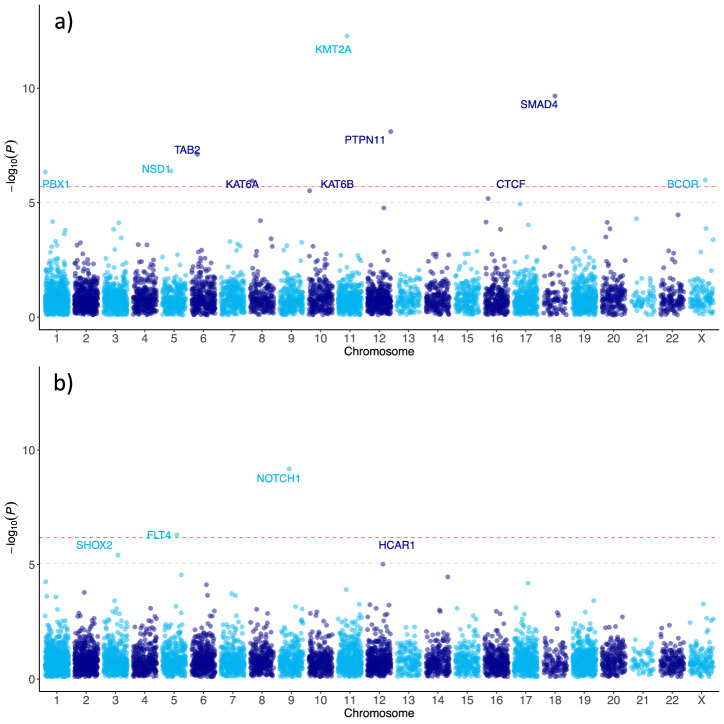
Log-transformed minimal *p*-value (*P*) per gene (y-axis) against its chromosomal location (x-axis). Red dashed line denotes the threshold for genes reaching exome-wide significance (Bonferroni adjusted *P* < 0.05); grey dashed line marks the threshold for genes reaching suggestive exome-wide significance (FDR 5%). **a** Burden analysis of sCHD vs. controls; **b** burden analysis of nsCHD vs. controls.

Of the genes identified as significant in sCHD, *KMT2A* (AD Wiedemann-Steiner syndrome OMIM 159555) showed the highest enrichment (Fig. 3a). *NOTCH1* (AD Adams-Oliver syndrome 5, Aortic valve disease 1 OMIM 190198) showed the highest number of variations in the nsCHD cohort (Fig. 3b) and warranted further investigation (companion manuscript).

Other genes reaching a significant level of association included *NSD1* (AD Sotos Syndrome OMIM 606681), *TAB2* (AD Non-Syndromic CHD 2, OMIM 605101), *KAT6A* (AD Arboleda-Tham Syndrome OMIM 601408), *PTPN11* (AD Noonan Syndrome OMIM 176876), *SMAD4* (AD Mhyre Syndrome OMIM 600993), *FLT4* (AD Congenital heart defects, multiple types, 7 OMIM 136352), and the X-linked gene *BCOR* (XLD Syndromic Micropthalmia OMIM 300485). They have all been previously described in the context of CHD, and our results corroborate these findings. The relatively low number of variations observed in well-known syndromic CHD genes (e.g., *CHD7* and *KMT2D*) could be because most participants were non-syndromic, unlike in previous studies.

The association of *PBX1* (AD Congenital anomalies of kidney and urinary tract syndrome with or without hearing loss, abnormal ears, or developmental delay OMIM 176310), *CTCF* (AD Intellectual developmental disorder, autosomal dominant 21 OMIM 604167) and *KAT6B* (AD Genitopatellar syndrome and SBBYSS syndrome OMIM 605880) with CHD (Table 1) have been previously reported in single cases or small patient cohorts, and our results add further evidence for an association with CHD.

*HCAR1* (OMIM 606923) and *SHOX2* (OMIM 602504) have not previously been associated with CHD at a genome-wide level. However, both genes were significantly associated with nsCHD at *FDR* 5% (Fig. 3b).

We also performed the same gene-based burden analysis for homozygous and likely compound heterozygous protein-altering variants; however, no significant associations were detected (Supplementary Data 4).

### Differentially expressed genes in cardiac-specific cells show a distinct enrichment pattern in syndromic and non-syndromic CHD

Previous studies have revealed significant levels of expression in the heart of genes associated with CHD^7,8^. By using publicly accessible bulk RNAseq data^15^ (tissue expression for heart, kidney, brain, and liver in 4–8 week-post-conception, see Methods), we consistently showed that genes with significant level of association in our case-control analysis also showed high expression in cardiac tissues (Supplementary Fig. 5). Moreover, syndromic CHD genes showed a systematic elevated expression in brain and liver (Bonferroni corrected *P* < 0.05), compared to non-syndromic CHD (Supplementary Fig. 6). The difference in expression patterns between these two groups was negligible in the heart, though (*P* > 0.05, Wilcoxon test; Supplementary Fig. 6). Despite its relevance, bulk RNAseq data analysis does not stretch as far as the delineation of expression patterns at the cellular level.

**Fig. 4:**
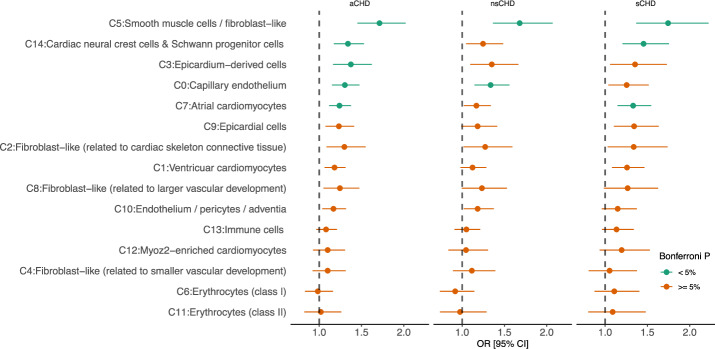
Logistic regression-based enrichment analysis of differentially expressed genes (DEGs) in cardiac-specific cell clusters for high-confidence loss-of-function variants (hcLOF). The analysis was stratified by syndromic status (aCHD, sCHD and nsCHD). The x-axis denotes the Odds Ratio (OR) and the 95% confidence interval. *P-*values were adjusted using the Bonferroni method (0.05/45 tests) to assess significant enrichment. Cardiac cell clusters C0, C3, C5, C7 and C14, show significant enrichment when analysing aCHD vs. controls. The enrichment observed in clusters C7 and C14 showed a major contribution of sCHD. In comparison, cluster C0 provided the major contribution to nsCHD.

To accomplish this, we assessed the mutational burden of rare non-synonymous variants (hcLOF and missC) within differentially expressed genes (DEGs) in cardiac-specific cells. We meta-analysed the exome data with a publicly available human heart transcriptomic dataset generated from early developmental stages of the human heart (6.5 and 7 weeks post-conception)^9^. Using the logistic regression framework mentioned above, we performed gene-set enrichment analysis on DEGs defined on 15 distinct cardiac cell clusters (C0-C14) reported by Asp et al.^9^. Both hcLOF and missC mutations were evaluated independently, and the analysis was stratified further by proband CHD status versus controls (aCHD, sCHD and nsCHD, Fig. 4).

Five cardiac-specific cell clusters were found significantly enriched (Bonferroni adjusted *P* < 0.05) for hcLOF variations when analysing aCHD probands vs. controls (Fig. 4): Smooth muscle cells (C5), Cardiac neural crest cells (C14), Epicardium-derived cells (C3), Capillary endothelium (C0) and Atrial cardiomyocytes (C7). Enrichment of hcLOF variants for DEGs in Smooth muscle cells (C5) showed a significant contribution to both sCHD and nsCHD. Cardiac neural crest cells (C14) and Atrial cardiomyocytes (C7) contributed to sCHD in the main, whereas the cluster of Capillary endothelium cells was significantly enriched in nsCHD versus controls (Fig. 4).

A similar enrichment pattern was observed for missC variants (Supplementary Fig. 7). In addition to the clusters previously enriched for hcLOF variants—Capillary Endothelium (C0), Smooth Muscle Cells (C5), Atrial Cardiomyocytes (C7), and Cardiac Neural Crest Cells (C14)—two additional cardiac-specific clusters, Endothelium/Pericytes (C10) and Fibroblasts (C2), showed a significant burden of missC variants in CHD cases compared to controls.

The synonymous variants set was used as a negative control and did not identify enrichment in any clusters evaluated (Bonferroni adjusted *P* > 0.05, Supplementary Fig. 8).

Together, these results provide valuable evidence regarding the possible mechanisms involved in the pathogenesis of CHD.

### Gene ontology (GO) enrichment analysis

To provide additional supporting evidence for our previous findings, we performed Gene ontology (GO) enrichment analysis to identify relationships between enriched genes in the case-control burden analysis (unadjusted *P* < 0.001; Supplementary Data 5) and biological processes. We analysed the set of nsCHD (*n* = 32) and sCHD (*n* = 49) genes independently, with the Enrichr tool^16^ (see Methods). The two gene sets were nearly mutually exclusive, with *NOTCH1* being the only gene overlapping between them.

The data suggested that genes identified in the nsCHD analysis are mainly associated with more specific cardiac development biological processes, with noteworthy contributions from *MYH6*, *NOTCH1* and *SHOX2* (Supplementary Fig. 9a). Top GO terms enriched in the sCHD analysis were related to the regulation of DNA transcription. The observed signal was driven by *CTCF, KAT6A, KAT6B, KMT2A, PBX1*, among others (Supplementary Fig. 9b).

## Discussion

In this study, we amassed 57,628 human exomes and conducted both a gene- and gene-set centred case-control burden analysis to increase our understanding of the genetic causes of CHD. After quality control at the sample and variant level, we provide a comprehensive CHD case-control cohort with unrelated and ancestry-matched individuals. Specifically, the availability of detailed phenotype data allowed us to explore the differences between syndromic and non-syndromic forms of CHD.

By utilising gene-level constraint information^10^, we investigated the contribution and properties of loss-of-function and missense constraint variants independently for all CHD cases, as well as syndromic and non-syndromic CHD independently. Like earlier comparable studies^6,7^, our results revealed a higher contribution of LOF variants to CHD compared to missense variants, confirming that this type of variation represents the largest driver. Subsequently, the analysis of syndromic cases revealed a higher burden of LOF mutations when compared with the non-syndromic cohort. This effect was mainly a result of the contribution of genes with a higher intolerance to loss-of-function variations. This same pattern was also observed when analysing the genes based on missense constraint.

We next assessed the contribution to CHD at the gene level by performing a gene-based case-control burden analysis. Our analysis revealed ten genes that reached genome-wide significant levels of association with CHD (*NSD1*^17^, *TAB2*^18^, *KAT6A*^19^, *PTPN11*^20^, *CTCF*^21^, *SMAD4*^22^, *FLT4*^23^, *NOTCH1*^24,25^, *BCOR*^26^ and *KMT2A*^27^). Previous studies have associated these genes as a cause of CHD, and our results confirm this association (Table 1). Furthermore, four candidate genes (*PBX1, SHOX2, KAT6B, HCAR1*) were found contributing to both syndromic and non-syndromic CHD at *FDR* 5%. To our knowledge, these genes have either not previously been associated with, or have only been briefly described in the context of CHD.

*PBX1* has been primarily associated with congenital abnormalities of the kidney and urinary tract (CAKUT)^28^; however, previous studies have reported isolated cases carrying de novo missense variations leading to syndromic CHD^28,29^. In line with these early reports, our analysis revealed a significant burden of missense constrained variants in *PBX1* in syndromic CHD patients (Table 1). It has also been demonstrated that deficiency of *Pbx1* impacts branchial arch artery patterning and results in the failure of cardiac outflow tract septation^30^. Interestingly, this gene was also found to be differentially expressed in Epicardium and Smooth muscle cells (Supplementary Fig. 10). Together, our findings suggest that *PBX1* contributes significantly to syndromic forms of CHD.

*SHOX2* was significantly enriched (at *FDR* 5%) in the nsCHD cohort, for missC variants (Table 1). Recent studies in animal models have demonstrated that the *Shox2* null mice are embryonic-lethal^31^. Cardiovascular defects identified in these mice included an abnormally low heartbeat rate, a severely hypoplastic Sinoatrial Node (SAN), hypoplastic or absent sinus valves^31^, and other atrial abnormalities (e.g. enlarged atrial chamber and thinner atrial wall). Subsequently, *SHOX2* has been described as playing a key role in developing the Sinoatrial Node^31,32^. In addition, *SHOX2* was identified as a significant DEG in atrial cardiomyocytes (Supplementary Fig. 10), providing further supporting evidence of its role in heart development, most likely by regulating the activity of *NXK2-5*^31,33^ and *TBX5*^34^. These results imply that *SHOX2* is a plausible novel non-syndromic CHD gene.

Truncating variants in the Lysine Acetyltransferase 6B gene (*KAT6B*) have been associated with Say–Barber–Biesecker–Young–Simpson Syndrome (SBBYSS, OMIM 603736) and Genitopatellar Syndrome (GTPTS, OMIM 606170). Heart defects have been reported as part of the phenotypic spectrum of SBBYSS^35^. In a recent study of 32 individuals with *KAT6B* disorder, 47% showed cardiovascular anomalies, mainly atrial septal defects, ventricular septal defects, and patent ductus arteriosus^36^. Our results have identified that *KAT6B* was differentially expressed in the cluster of atrial cardiomyocytes cells (Supplementary Fig. 10), which suggests a possible role in the early cardiac development programme. Our analysis extends previous findings associating loss-of-function variations in *KAT6B* to sCHD.

The Hydroxycarboxylic Acid Receptor 1 *(HCAR1)* is not known to be involved in cardiac development or CHD, and a query of major transcriptomic data resources, such as GTEx, Expression Atlas, and the Human Protein Atlas, showed negligible or absent expression in human and mouse heart tissue. We did not observe enrichment or differential expression in any cardiac cell clusters analysed in this study. Published knockout and CRISPR screen data also provide no functional link to the heart. While our data highlight *HCAR1* as a putative candidate for CHD, its biological role in cardiac contexts remains uncertain and warrants further investigation.

Supplementary Data 6 provides an overview of the cardiac sub-phenotype spectrum observed among carriers of the top significant genes identified in this study.

By meta-analysing the genomic data with heart single-cell transcriptomic data, we investigated the pattern of expression of DEGs for aCHD, sCHD and nsCHD in a range of cardiac-specific cells. Using Gene Ontology enrichment as a complementary analysis, we identified key gene markers and biological processes associated with CHD. Unlike previous studies^7,37^, which focused on whole heart bulk-RNA sequencing data, the use of transcriptomic data at a single-cell resolution allowed the analysis of candidate gene expression patterns in specific cardiac cell clusters important for early cardiac development. Our analysis highlighted distinct cardiac cell clusters contributing to sCHD and nsCHD. In addition, we demonstrated that missense constrained variants could have a similar functional impact compared to loss-of-function variants, although to a lesser degree. For instance, the significant enrichment of sCHD in cardiac neural crest cells (cNCCs) suggests a broader contribution of patients affected by syndromic occurrences, not limited to heart development only. Perturbations in the cNCCs migration process can lead to a wide spectrum of human cardio-craniofacial syndromes, including DiGeorge Syndrome (22q11.2 Deletion Syndrome, OMIM 188400) and CHARGE (OMIM 214800). The enrichment observed in capillary endothelium and pericyte cells in nsCHD, associated with the vasculogenesis process, suggests that the phenotypic occurrence in these patients is limited to the cardiovascular system rather than affecting a broader spectrum of cells.

Whilst the results are promising, they are limited because the currently available human heart single-cell map^9^ covers only 6.5–7 post-conception weeks, leaving earlier cell lineages (e.g. left/right and early cardiomyocyte progenitors) unrepresented. Thus, some early-acting cell types relevant for the developing heart may be underestimated, and enrichment should be interpreted within this developmental window. Although mouse single-cell datasets at earlier developmental stages are available, cross-species integration requires careful harmonisation of developmental times, ortholog mapping, and analytical frameworks. Such methodological considerations extend beyond the scope of the present study, but they represent an important direction for future work.

Preliminary digenic analyses were explored but not considered for inclusion in the present work after extended validation revealed non-specific enrichment patterns, underscoring the need for more robust statistical frameworks to accurately evaluate oligogenic contributions to CHD.

This study has limitations inherent to large-scale case-control exome designs. Our cohort was predominantly of European ancestry (~92%), with limited representation from other ancestries, which constrains the generalisability of allele frequency estimates and genetic associations. In addition, the control cohort, derived from the UK Biobank, consists mostly of adults, whereas a substantial proportion of CHD cases were enrolled in childhood. This age imbalance introduces a potential survivorship bias: rare variants associated with severe CHD subtypes may be underrepresented in controls, which could impact effect sizes for some gene-disease associations. The syndromic/non-syndromic classification may be incomplete for individuals enrolled at a young age, before neurodevelopmental features can be fully assessed, although for a large proportion of cases, temporal data across multiple clinical visits were available. Thus, the stratified burden analysis results, particularly the non-syndromic stratum, should be interpreted taking into account this caveat. Of note, the primary unstratified analysis (all CHD cases versus controls) is independent of this classification. Label-permutation testing confirmed that modest residual genomic inflation (lambda_1000 = 1.04–1.05) exceeds what the case-control imbalance alone explains, most likely reflecting sub-continental European allele frequency differences between the predominantly German case and predominantly British control cohorts, as well as residual capture platform effects. Importantly, nine of the ten Bonferroni-significant genes are well-established CHD genes, and *PBX1* has prior functional evidence supporting a role in cardiac development, indicating that the observed gene-level burden signals are not driven by technical or population artefacts. (Supplementary Information).

In summary, we analysed ~57,000 exomes, and complemented this with transcriptomic data at single-cell resolution. The findings have strengthened the association of previously described genes with CHD, identified novel candidate genes, and provided a deeper understanding of the pathophysiological mechanisms underlying CHD at the gene level and the potential different aetiologies between syndromic and non-syndromic CHD.

## Methods

### Ethics statement

This study was conducted in accordance with the Declaration of Helsinki. Analyses were performed on de-identified human genomic data obtained through approved access to controlled-access resources. Access to congenital heart disease (CHD) case data was granted by the European Genome-phenome Archive (EGA) Data Access Committees following protocol review. The datasets used in the study had prior institutional research ethics board approval from the German Heart Registry (Berlin, Germany), the University of Leuven, Belgium (number: B322201010111/S52853), and the University of Nottingham by the East Midlands-Leicester Research Ethics Committee (number 6721). The DDD study was approved by the UK Research Ethics Committee (10/H0305/83, granted by the Cambridge South Research Ethics Committee and GEN/284/12, granted by the Republic of Ireland Research Ethics Committee). UK Biobank whole-exome sequencing data were accessed under approved application number 44165.

### Cohort description

To create a comprehensive CHD case-control cohort, exome sequencing data from multiple individuals was combined in a unique reference dataset. CHD cases were mainly sequenced as part of an initiative from the German Competence Network for Congenital Heart Defects, the Deciphering Developmental Disorder (DDD) project and the University of Nottingham (UK); controls were sequenced as part of the UK Biobank (UKBB). Samples from the UKBB dataset with phenotype description labelled as Schizophrenia (SCZ), bipolar disorder (BP), developmental delay (DD) or other congenital anomalies were excluded from the analysis. Accordingly, a small fraction of samples in the UKBB cohort (127 samples), labelled as CHD cases, were included in the analysis. In total, we assembled an exome dataset consisting of 57,628 samples (4747 CHD cases and 52,881 controls). Clinical data on cardiac and extracardiac features were extracted from electronic health records (EHRs) and standardised using Human Phenotype Ontology (HPO) terms (Supplementary Data 1). The syndromic/non-syndromic classification was assigned based not only on the presence of extracardiac features, including dysmorphic features, growth abnormalities, and recognised genetic syndromes, but also taking into account multiple clinical reports accessible through constantly updated registry data across different clinical visits.

### Alignment, variant calling, quality control and variant annotation

The assembled dataset was processed and harmonised using the same alignment (BWA v0.3), calling (GATK v4.0), annotation (VEP v95) and quality control (Hail v0.2) pipelines. Supplementary figures and tables providing supporting quality-control diagnostics are included in the Supplementary Information (Supplementary Figs. 11–18 and Supplementary Tables 1–7).

### Defining a set of loss-of-function and missense constraint variants

We enriched the dataset for high-confidence loss-of-function (hcLOF) variants and missense constrained (missC) variants. hcLOF variants were annotated as indicated by the LOFTEE tool (https://github.com/konradjk/loftee) with its default parameters and included stop-gained, essential splice and frameshift variants. To define a set of missC variants, we evaluated four state-of-art pathogenicity prediction scores: CADD^38^, MPC^39^, REVEL^40^ and MVP^41^. Specifically, the performance of these scores was assessed by classifying benign and pathogenic missense variants (accessed through the ClinVar database, https://www.ncbi.nlm.nih.gov/clinvar) in the context of known CHD genes. In brief, receiver operating characteristic (ROC) analysis was conducted for benign and pathogenic variants within known CHD genes. The analysis was further stratified by splitting the gene set into LOF constraint (LOEUF < 0.35) and LOF non-constraint (LOEUF > = 0.35) genes. A score was defined as a ‘good predictor’ if it achieved an area-under-curve (AUC) > 90% in both evaluated scenarios. Three of these scores: CADD (threshold = 24), REVEL (0.5) and MVP (0.8) met this criterion. A missense variant was defined as missC if it was predicted as likely deleterious by at least two of these scores based on the optimal threshold suggested by the ROC analysis (Supplementary Fig. 3).

### Defining rare variants

Variants were filtered based on the cohort-specific allelic frequency (‘internal’ AF) as well as using external datasets. A variant was defined as rare if AF was lower than 0.001 (MAF < 0.001) in the gnomAD database^10^ (both exomes v2.1.1 and genomes v3.0.0), the RUMC cohort^42^, as well as AFs from an in-house German exome sequencing cohort.

### Gene-set enrichment analysis

#### Generation of gene sets

Gene set-level association analysis was performed to assess whether an excess of the possible pathogenic variants was enriched for a particular category of genes (as described below). This procedure was executed for the following gene sets:

- LOEUF gene bins: Constraint loss-of-function (LOF) metrics per protein-coding genes were accessed through gnomAD resource^10^. Genes were ranked by their observed/expected LOF mutation ratio upper fraction (termed LOUEF), and ten bins with an equal number of genes (~1900 genes per bin) were defined. Lower values of LOEUF (e.g., bins 1 and 2) denote most LOF-constrained genes.
- MOEUF gene bins: Similar as described above for LOEUF genes, but genes were binned based on their observed/expected missense mutation ratio upper fraction (termed MOEUF).
- Differentially expressed genes (DEGs) in cardiac-specific cells: DEGs identified in 15 distinct cardiac cell clusters reported by Asp et al.^9^. In brief, genes were determined as significantly differentially expressed in a particular cardiac cell cluster if the averaged log-fold change (logFC) > 0 (upregulated) at FDR 1%.

#### Gene set-based association analysis

For each sample within the filtered dataset, we generate a Minimal Allele Count (MAC) metric by aggregating high confidence Genotypes (DP > = 10, GQ > = 20 and allelic balance heterozygous > 0.2) across the genes within the gene set. Then, a burden logistic regression test was performed using CHD case/control status as response and the first five ancestry principal component and sex as covariates using the Hail function *hl.logistic_regression_rows*. The analysis was stratified at the sample and variant level. At the sample level, the data was divided based on the syndromic status; three categories were tested: aCHD (all CHD cases vs. control), nsCHD (non-syndromic CHD cases vs. control) and sCHD (syndromic CHD cases vs. control). At variant level, three different groups were evaluated based on the predicted severity of the variants: hcLOF (most severe), missC and synonymous. The synonymous variant set was used as a negative control set at the variant level to evaluate for potential artefacts. The odds ratio (exp (beta coefficient)), 95% confidence interval and *p*-value metrics were used to evaluate significant enrichment.

### Gene-based burden testing

We performed case-control gene-centred burden test analysis to assess genes with significant association with CHD. Fisher Exact test was performed independently for rare (MAF < 0.001) hcLOF and missC variants. To define the significant study-wide *p-*value, the minimal *p-*value (*P*) per gene between these two categories was chosen. The analysis was further stratified by syndromic status to assess the distinct contribution of these categories to CHD. A gene was defined as genome-wide significant if it reached a Bonferroni corrected *P* < 0.05, using a correction factor of 2 × 16,351 tests to account for testing two variant categories (hcLOF and missC) per gene (total tests = 32,702). False discovery rate (FDR) adjustment using the Benjamini–Hochberg method was applied across the same total number of tests. In addition, the set of synonymous variants was used as a negative control set since no difference between cases/control is expected on this set of variations (quantile-quantile plots, Supplementary Fig. 4). A subset of cases in this cohort may carry pathogenic copy-number variants identified through an ongoing whole-genome sequencing study. By design, individuals selected for genome sequencing were exome-negative; i.e., they did not carry rare damaging SNVs or indels in genes reaching significance in the present burden analysis; and therefore do not contribute to the gene-level burden signals reported here. Additional calibration analyses, including MAF-stratified evaluation of synonymous variant test statistics and label-permutation null tests, were performed to characterise residual genomic inflation (Supplementary Information, Supplementary Figs. 19, 20 and Supplementary Tables 8, 9).

### Expression analysis using bulk RNAseq data

A publicly available human transcriptomic dataset previously described by Cardoso-Moreira et al.^15^ was used to complement this study. To assess the gene expression levels in the heart, kidney, brain, and liver; the RPKM matrix hosted in ArrayExpress (E-MTAB-6814) was used. Gene expression levels were averaged among samples in the early developmental stages (4–8 weeks-post-conception). Percentile rank per gene was computed based on the mean expression.

### Gene ontology enrichment analysis

The R-package *Enrichr*^16^ was used to perform GO enrichment analysis, against the *Biological_Process_2023* database using ‘all genes’ as background. The analysis was conducted on the set of sCHD (*n* = 49) and nsCHD (*n* = 32) genes, which showed an unadjusted *P* < 0.001 (Fisher Exact test) in the case-control burden analysis. GO terms with only one overlapping gene were not considered. Results are summarised in Supplementary Data 5.

## Supplementary information

Supplemental_Information_042026

Supplementary_Data_1_Cohort_metadata

Supplementary_Data_2_Geneset_enrichment_analysis

Supplementary_Data_3_Gene_enrichment_analysis

Supplementary_Data_4_Recessive_analysis

Supplementary_Data_5_GO_enrichment_analysis_sCHD_nsCHD

Supplementary_Data_6_Top_Genes

## Data Availability

The CRAM-level data from CHD patients used in this study can be accessed under the following accession codes (European Genome-phenome Archive): EGAD00001002200, EGAD00001000796, EGAD00001000797, EGAD00001000800, EGAS00001000544, EGAS00001000775, EGAS00001000762. UK Biobank 50K WES dataset freeze was accessed under the application number 44165.

## Code availability

Pipelines for sample/variant quality control (QC), annotation and burden testing are available on GitHub: https://github.com/enriquea/wes_chd_ukbb.
