# Supplementary material for: Assessing the contribution of rare variants to congenital heart disease through a large-scale case-control exome study
Source: NPJ Genom Med. 2026 Jun 2;11:30. doi: 10.1038/s41525-026-00582-z (PMC13230774; doi:10.1038/s41525-026-00582-z)
Supplement: Supplementary file 1 — Supplemental_Information_042026 [file 41525_2026_582_MOESM1_ESM.pdf]

Supplementary Information

Table of Contents

Supplementary Figures ***2***
Alignment and variant calling ***14***
Sample QC ***15***
Hard filters. **15**
Inferring population ancestry **16**
Inferring sample relatedness **19**
Platform inference. **20**
Platform- and population-specific outliers filtering. **21**
Final sample QC and evaluation. **22**
Variant QC ***24***
Hard filters. **24**
RF model. **25**
VQSR filter. **26**
Coverage. **27**
Variant annotation ***29***
Calibration of gene-based burden tests ***29***
References ***33***

Supplementary Figures

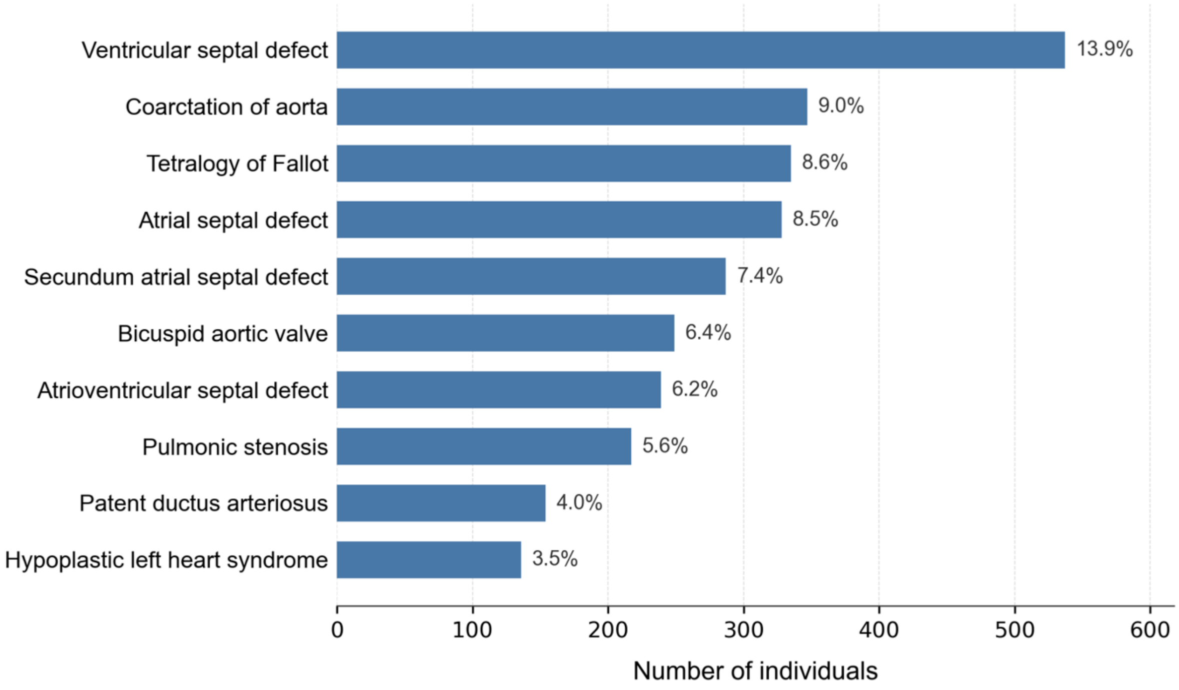

Supplementary Figure 1. Top 10 most frequent cardiac phenotypes in the QC-passed CHD cohort. Bars show the number of individuals annotated with each phenotype term, with major contribution of septal defect forms (>30%). Individuals may present with multiple cardiac phenotypes and can therefore contribute to more than one category. Percentages are relative to the total number of cases.

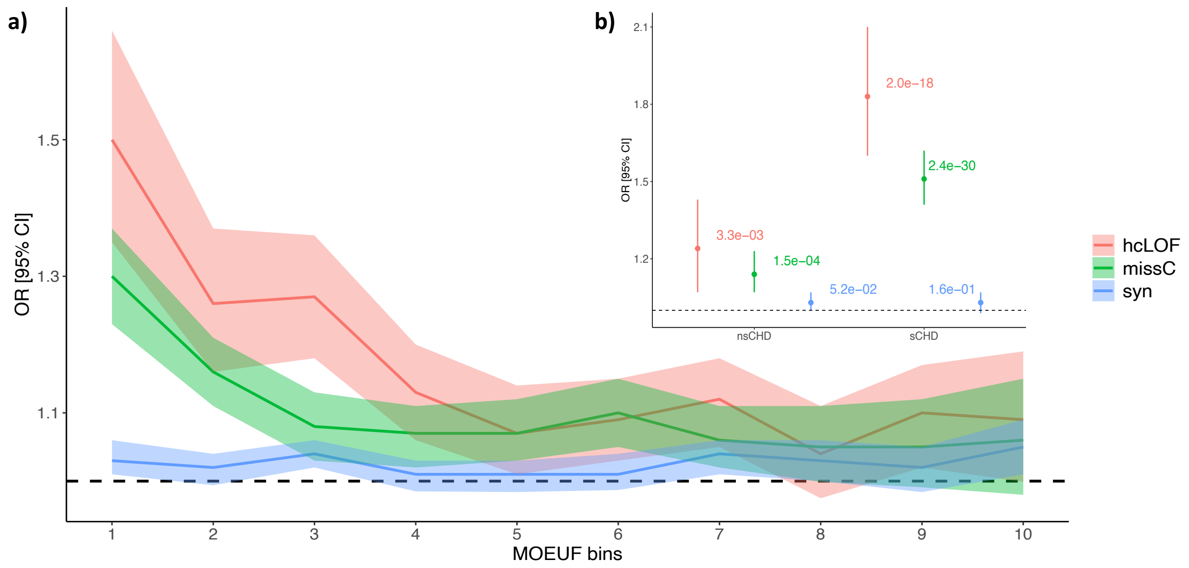

Supplementary Figure 2. a) Enrichment analysis across the missense constraint gene spectrum (aCHD vs controls). Protein-coding genes were binned based on the MOEUF metric as proposed by gnomAD. Every bin contains ~1,900 genes. Top bins (1, 2) contain the genes with the highest intolerance to missense variation. **b)** Enrichment analysis stratified by syndromic status (sCHD and nsCHD) vs controls in the top constraint MOEUF bin (1). The x-axis indicates the constraint bins; the y-axis shows the Odds Ratios (OR) and the 95% confidence interval. hcLOF: high-confidence loss-of-function variants; missC: missense constrained variants; syn: synonymous variants.

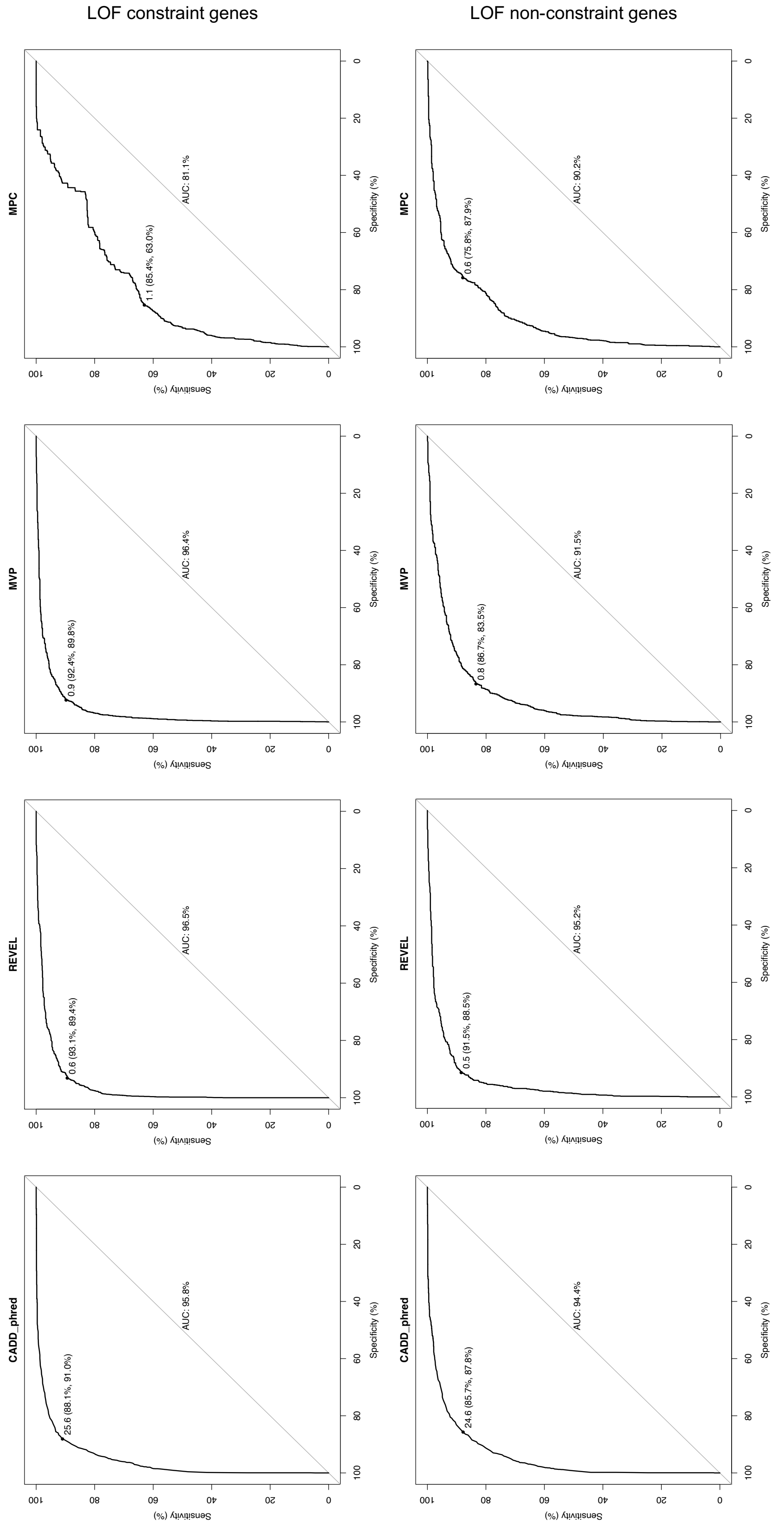

(**Supplementary Figure 3.** Legend on next page)

Supplementary Figure 3. ROC analysis of pathogenicity prediction scores (CADD, REVEL, MVP and MPC). The analysis was performed on a balanced set of benign (true negative) and likely pathogenic (true positive) variants from the ClinVar database within known CHD genes. The top panels show the results for LOF constraint genes (LOEUF < 0.35). The bottom panels show the results for LOF non-constraint genes (LOEUF >= 0.35).

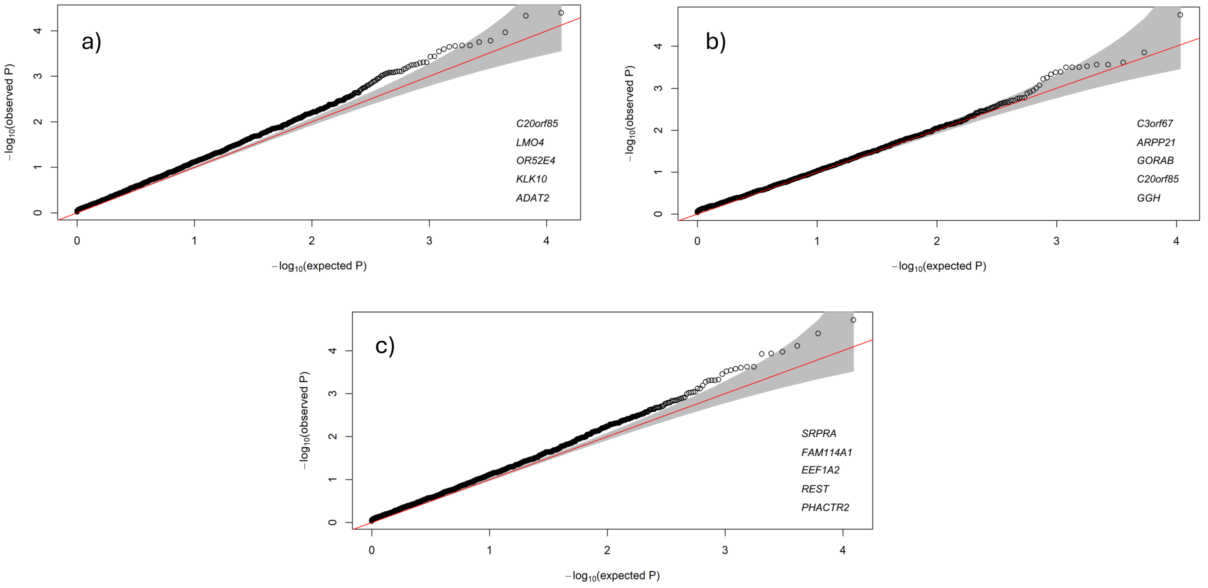

Supplementary Figure 4. Quantile-quantile plots. Expected vs observed p-values for synonymous variants stratified by syndromic status (MAF < 0.001). (a) aCHD vs controls, raw lambda: 1.3455, adjusted lambda_{1000}: 1.0398. b) sCHD vs controls, 1.3513, 1.0404. c) nsCHD vs controls, 1.4731, 1.0545. Top five enriched genes are listed.

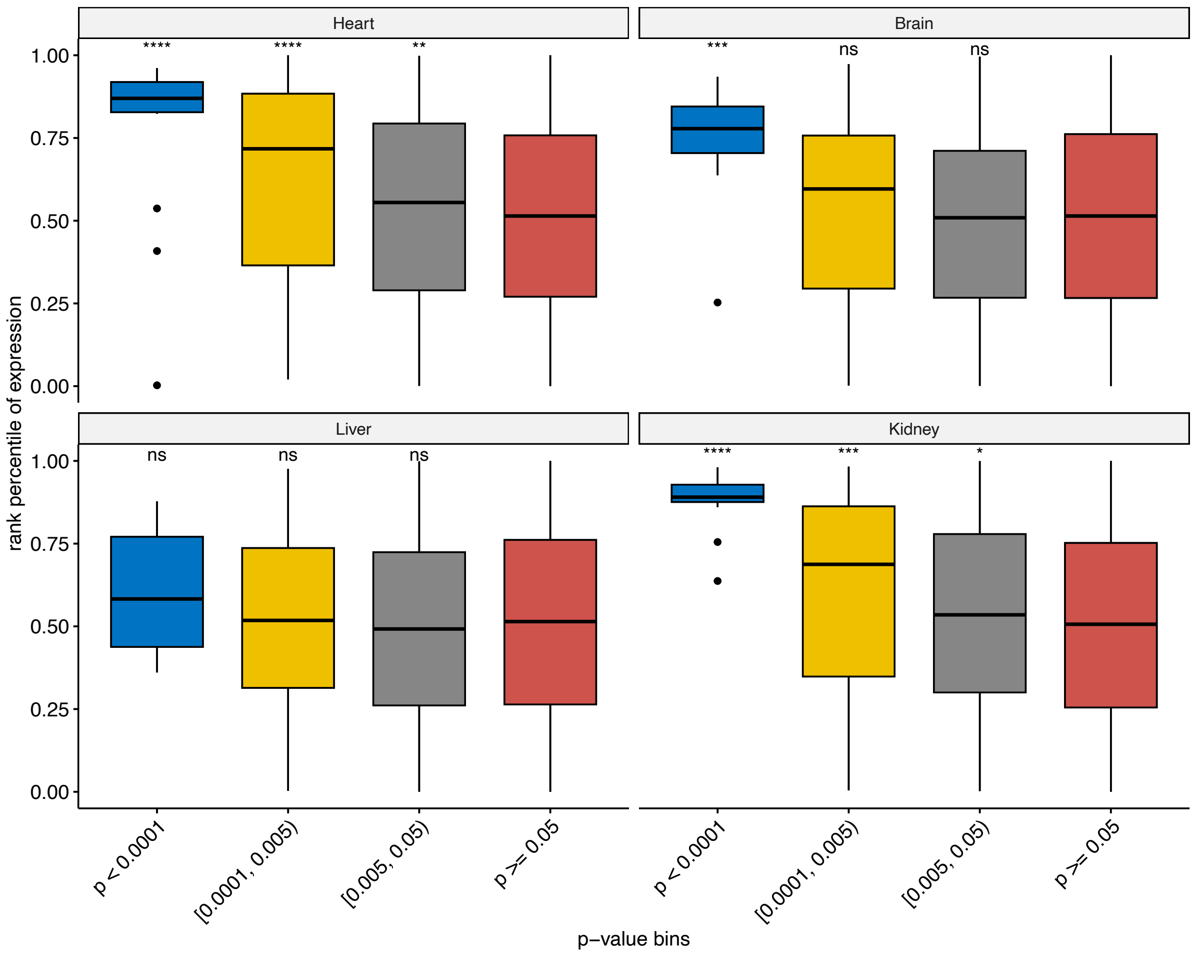

Supplementary Figure 5. Expression pattern of CHD genes in different tissues (Heart, Brain, Liver and Kidney). X-axis denotes gene p-value bins. P-value refers to the minimal p-value (*P*) observed in the gene-based enrichment analysis for rare hcLOF and missC variants. The gene association analysis was performed by comparing all CHD probands (aCHD) vs controls. Y-axis denotes tissue-specific percentile rank of mean expression. Averaged expression was computed for samples between 4-8 weeks-post-conception (developmental stage). More significant genes (blue box) in the CHD case-control analysis showed the higher expression rank (e.g., Heart, Brain, and Kidney). Mean comparisons between bins were computed using the Wilcoxon test (alternative: greater; reference group (red box): genes with $P > 0.05$ in the case-control analysis). ns: p > 0.05; *: p <= 0.05; **: p <= 0.01; ***: p <= 0.001; ****: p <= 0.0001.

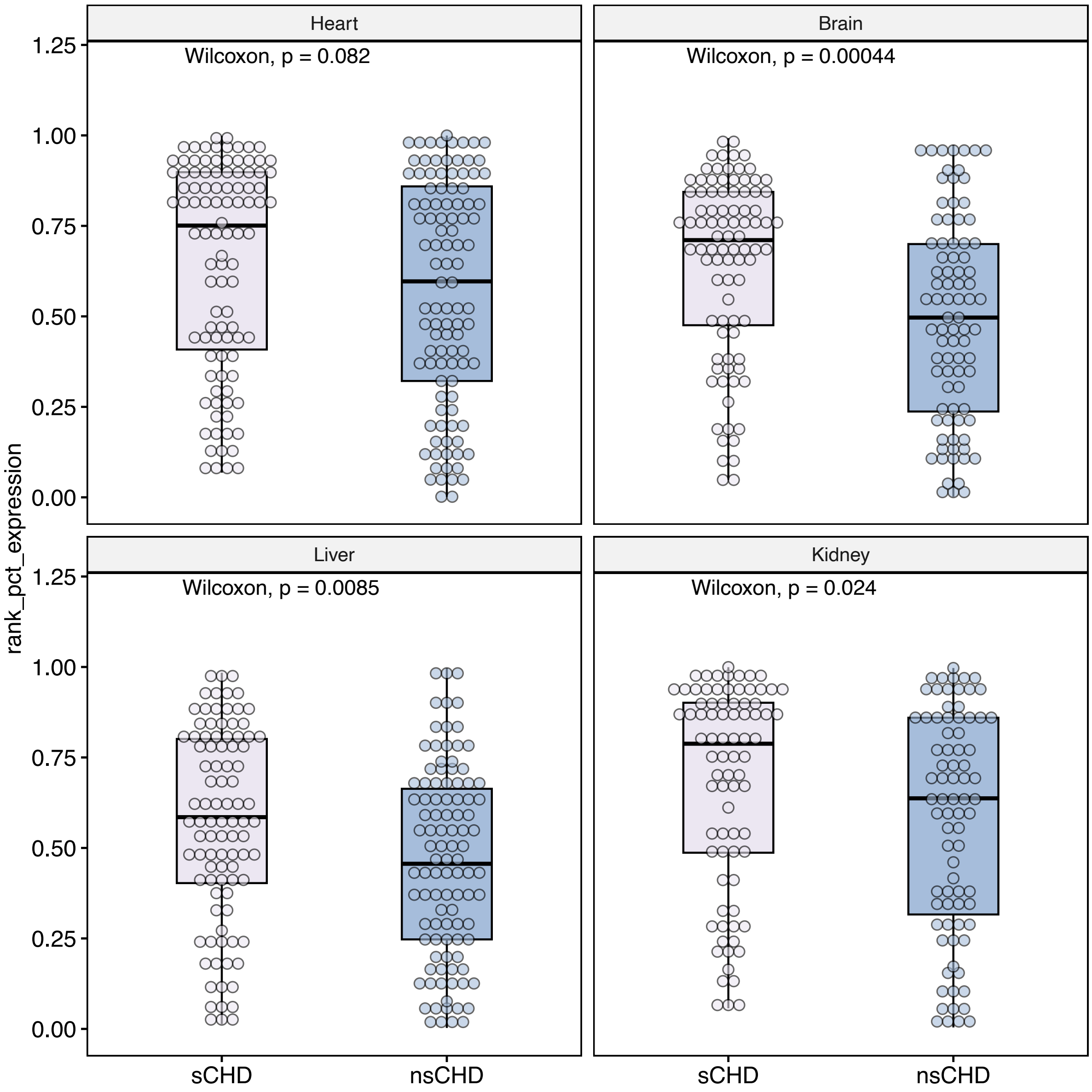

Supplementary Figure 6. Tissue-specific expression pattern of CHD genes identified in the case-control analysis, stratified by syndromic status (syndromic (sCHD) and non-syndromic (nsCHD) vs controls). Only genes with unadjusted $P < 0.005$ in the case-control analysis are included. X-axis denotes the probands used in the case-control analysis (sCHD or nsCHD vs controls). Y-axis denotes tissue-specific percentile rank of mean expression. Averaged expression was computed among samples between 4-8 weeks-post-conception (developmental stage). Mean comparisons between groups were computed using the Wilcoxon test (two-sided). No significant difference was observed in the heart for sCHD and nsCHD genes ($P > 0.05$). After correction (0.05/4), sCHD genes showed significant increased expression levels in brain and liver, compared to nsCHD (Bonferroni adjusted $P < 0.05$).

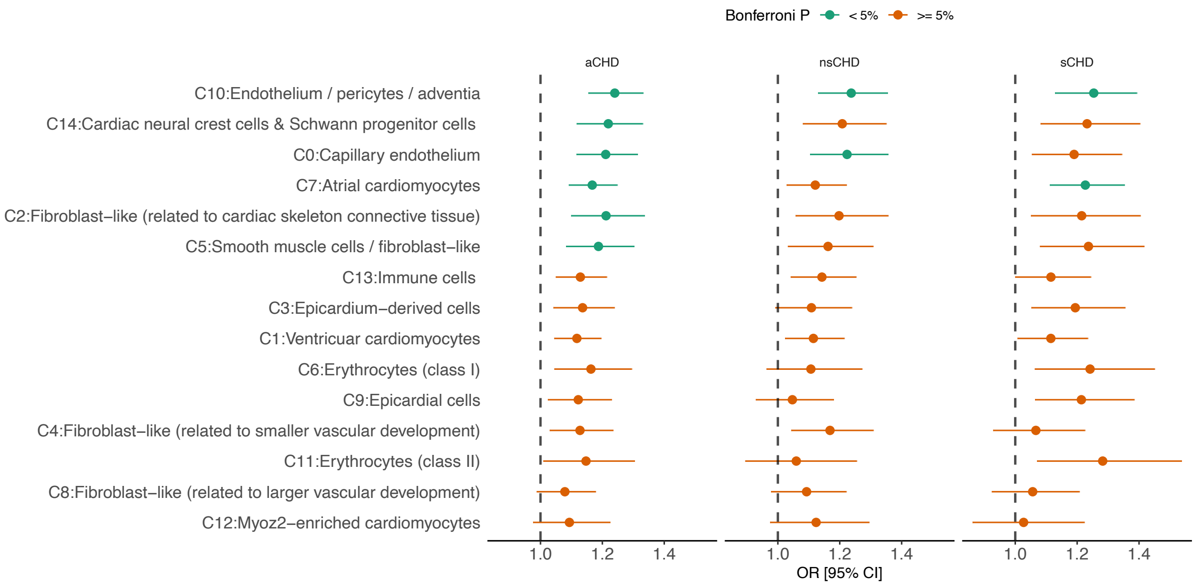

Supplementary Figure 7. Logistic regression-based enrichment analysis of differentially expressed genes (DEGs) in cardiac-specific cell clusters for missense constrained variants (missC). The analysis was stratified by syndromic status (aCHD, sCHD and nsCHD). The x-axis denotes the Odds Ratio (OR) and the 95% confidence interval. *P-values* were adjusted using the Bonferroni method (0.05 / 45 tests) to assess for significant enrichment.

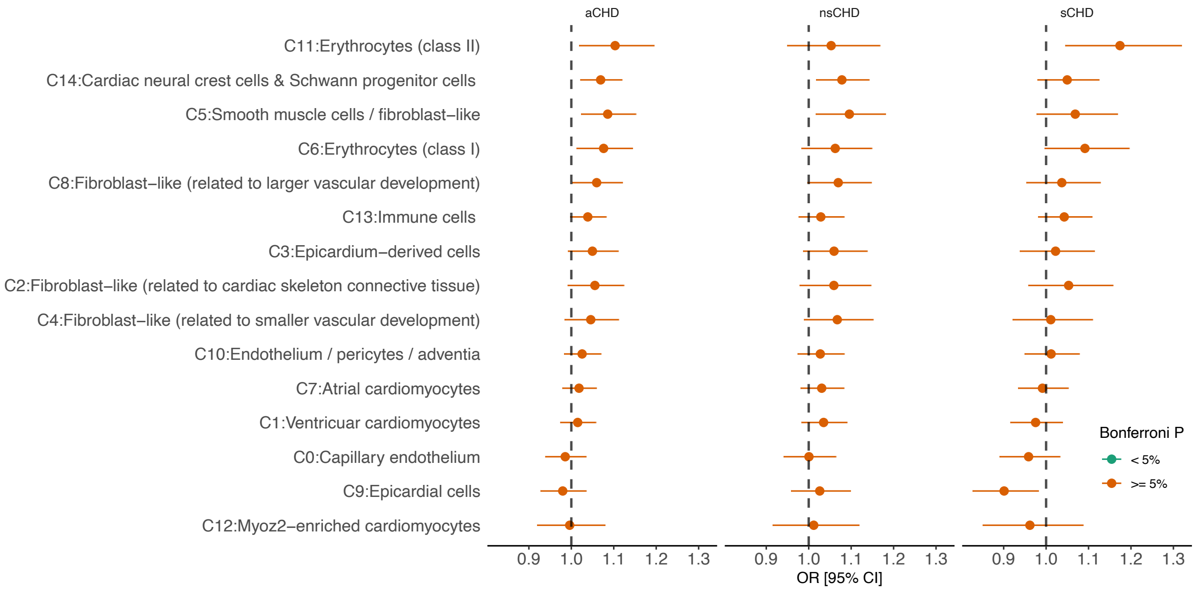

Supplementary Figure 8. Logistic regression-based enrichment analysis of differentially expressed genes (DEGs) in cardiac-specific cell clusters for synonymous variants. The analysis was stratified by syndromic status (aCHD, sCHD and nsCHD). The x-axis denotes the Odds Ratio (OR) and the 95% confidence interval. *P-values* were adjusted using the Bonferroni method (0.05 / 45 tests) to assess for significant enrichment.

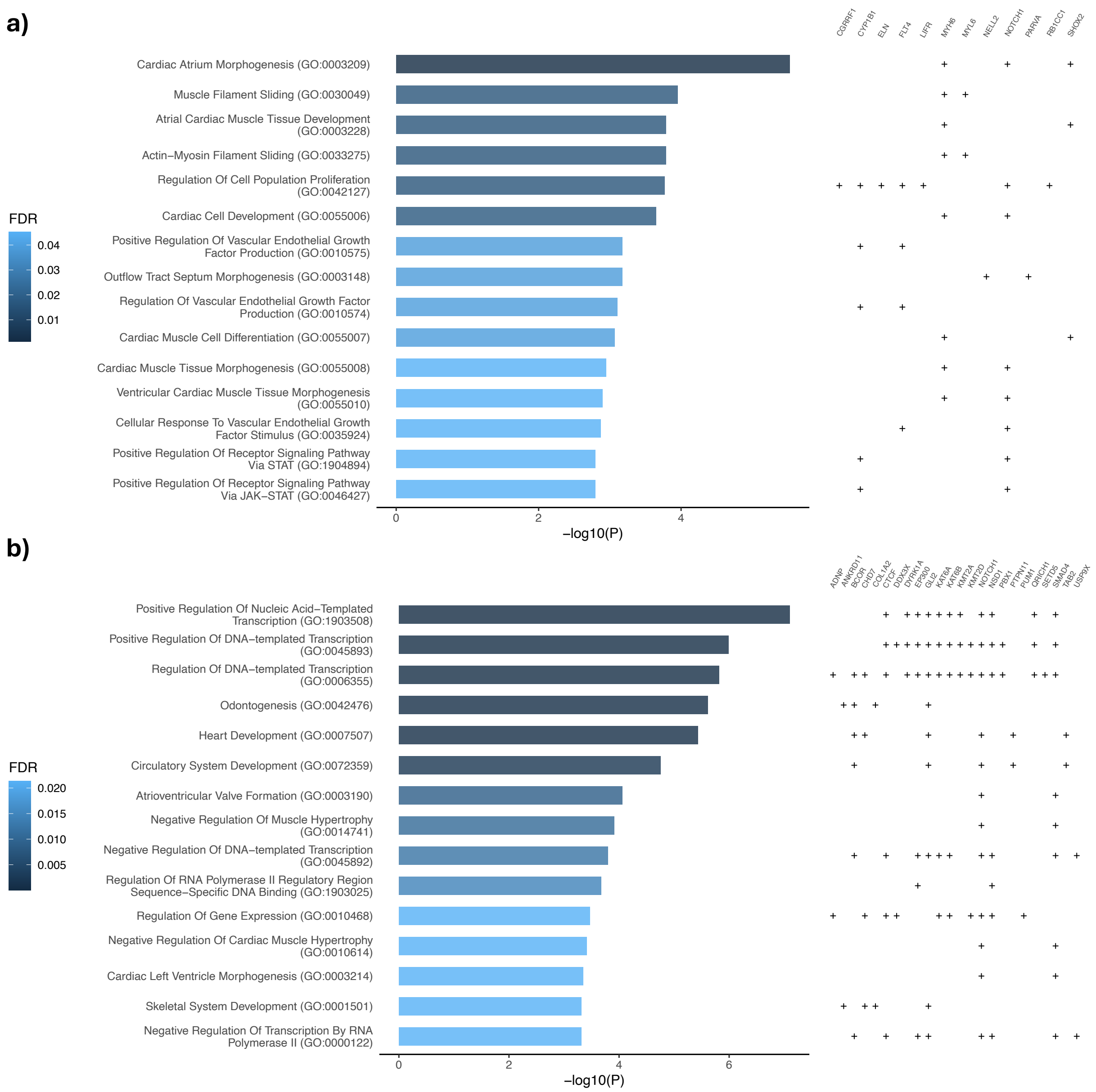

Supplementary Figure 9. Top Gene Ontology (GO) terms enriched for nsCHD and sCHD genes. The analysis was performed on the set of genes with unadjusted $P < 0.001$ in the case-control burden test. a) GO enriched terms for nsCHD genes (n=32). b) GO enriched terms for sCHD genes (n=49). Only *NOTCH1* was found overlapping between sets. For every GO term, the related genes (+) are shown. GO enrichment analysis was performed using the Enrichr tool, against Biological Process 2023 database using 'all genes' as background.

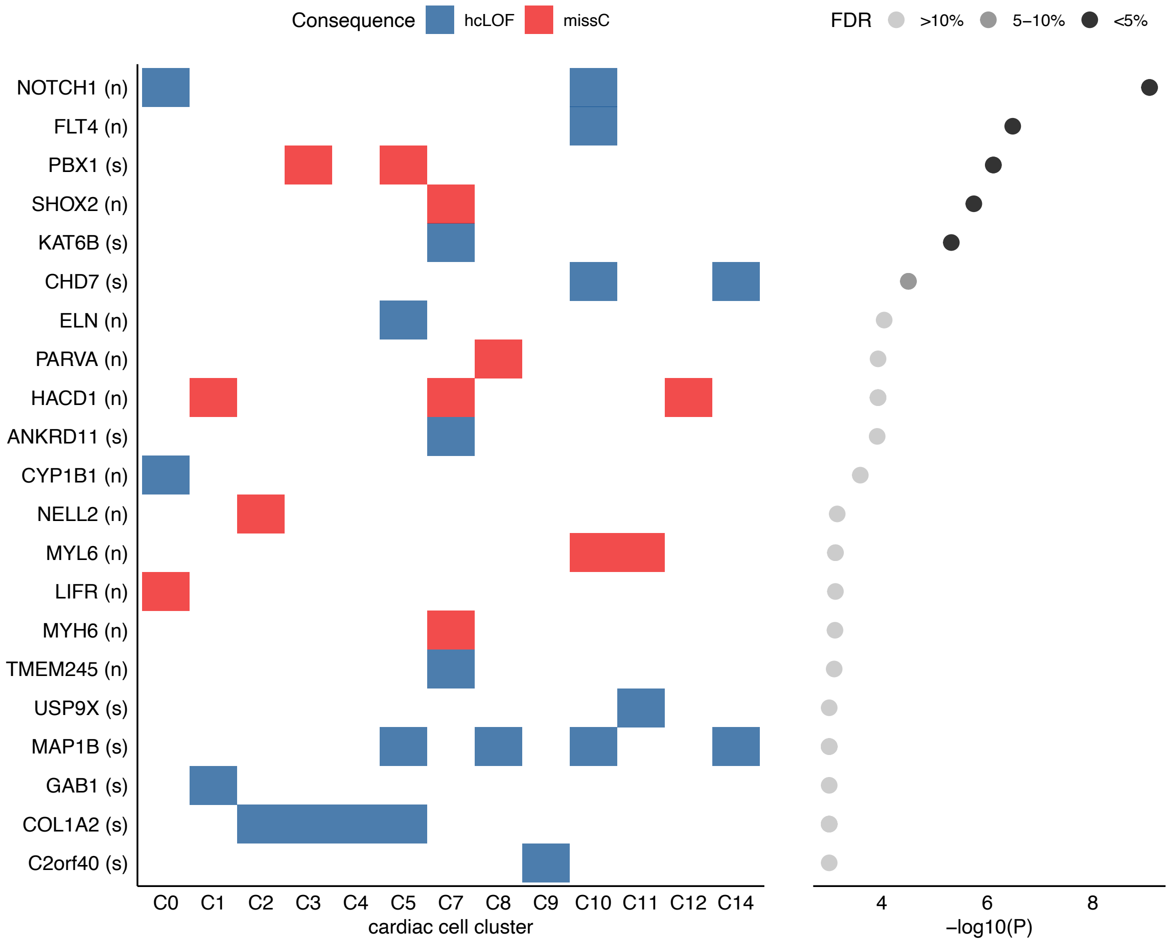

Supplementary Figure 10. Top enriched genes (unadjusted $P < 0.001$, case-control Fisher Exact test) found differentially expressed in at least one cardiac-specific cell cluster. The left plot shows the gene/cluster overlap and highlights the variant category with the highest enrichment (blue: hcLOF, red: missC). The x-axis denotes de cardiac clusters; the y-axis indicates the genes and the CHD category analysed (s: sCHD, n: nsCHD). The right plot shows the log-transformed *P* (x-axis) and the *FDR* significant level per gene. Six genes showed $FDR < 10\%$: *NOTCH1, FLT4, PBX1, SHOX2, KAT6B* and *CHD7*. C0: Capillary endothelium, C1: Ventricular cardiomyocytes, C2: Fibroblast-like (related to cardiac skeleton connective tissue), C3: Epicardium-derived cells, C4: Fibroblast-like (related to smaller vascular development), C5: Smooth muscle cells, C7: Atrial cardiomyocytes, C8: Fibroblast-like (related to larger vascular development), C9: Epicardial cells, C10: Endothelium/pericytes/adventia, C11: Erythrocytes (class II), C12: Myoz2-enriched cardiomyocytes, C14: Cardiac neural crest cells & Schwann progenitor cells.

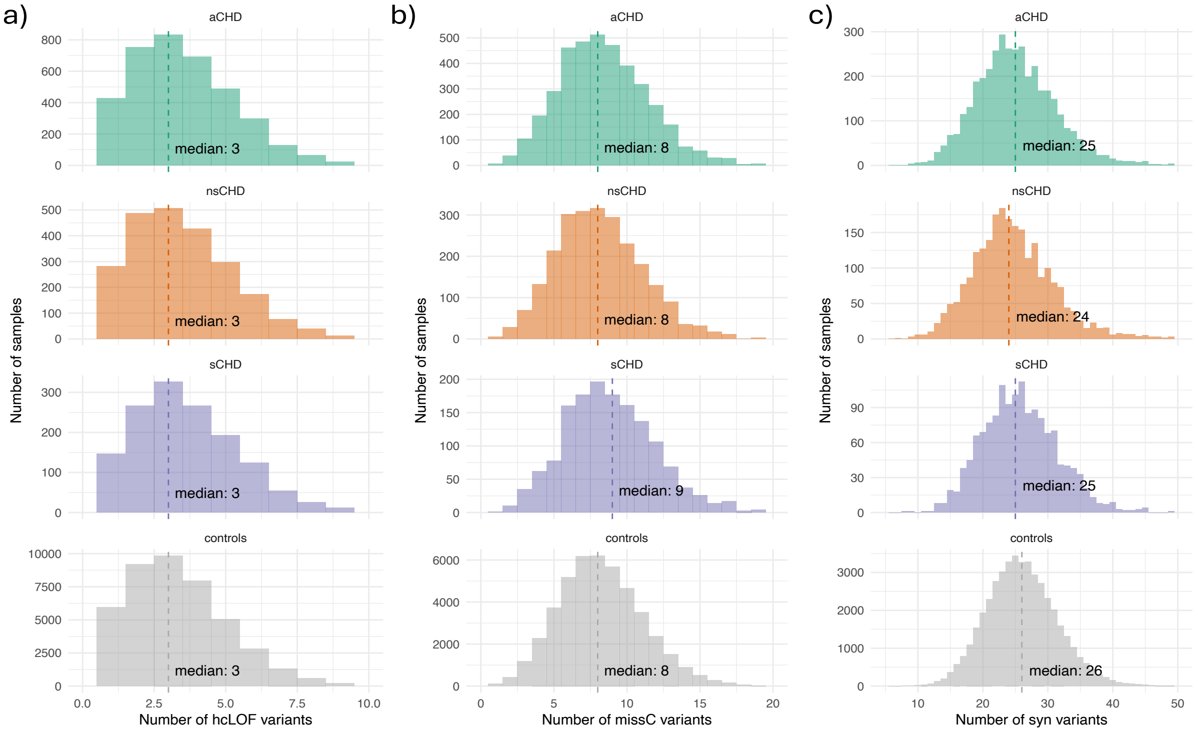

Supplementary Figure 11. Distribution of rare variants per samples stratified per phenotypes and variant type. a) High-confidence loss-of-function variants (hcLOF). b) Missense constrained variants (missC). c) Synonymous variants. sCHD: syndromic CHD cases; nsCHD: non-syndromic CHD cases; aCHD: all CHD cases.

Alignment and variant calling

CRAM-level data for all previously and newly sequenced samples were realigned to the human genome build GRCh38 using the BWA tool (version 0.7). Variants were jointly called using the Genome Analysis Toolkit (GATK, version 4.1), following the Broad Institute best-practice guidelines for germline single nucleotide variants (SNVs) and short insertions/deletions (indels). Briefly, HaplotypeCaller was used in GVCF mode to process samples individually, such that every position in the genome was assigned with a likelihood of being or not being a variant. The GenomicsDB (https://github.com/Intel-HLS/GenomicsDB) tool was used then to import and merge the per-sample GVCF genotype data. Samples were then jointly genotyped for high confidence alleles using the GenotypeGVCFs tool. The Variant Quality Score Recalibration (VQSR) in GATK was applied independently for SNVs and indels to assess variant call accuracy. The complete process was executed using standard pipelines from the Human Genetics Informatics (HGI) unit at the Wellcome Trust Sanger Institute (WTSI).

To perform scalable downstream analysis of the sequencing data, the multi-sample cohort-VCF generated from the previous step was imported into Hail 0.2 (https://hail.is), a python-like library for analysing genomic data at scale, using the function *hl.import_vcf.* Subsequent sample- and variant-level quality control (QC) was performed using the Hail framework (see below), following mainly the workflows proposed by the gnomAD project[1], otherwise explicitly specified. The Hail-based pipelines used in this study are publicly available on GitHub (https://github.com/enriquea/wes_chd_ukbb).

Sample QC

Hard filters.

To compute sample QC metrics, a set of high-confidence variants was defined by applying the following criteria: (i) bi-allelic, (ii) variants with high call-rate (> 0.99) across all samples in the call set and (iii) common single nucleotide variants (allelic frequency > 0.1%). The individual's chromosomal sex was inferred by calculating the inbreeding coefficient (*F-stat*) on chromosome X over the set of variants described above. The *hl.impute_sex* Hail function was used to perform the computation. This approach adopts the same implementation as the PLINK tool (v1.7). In addition, the coverage of the chromosome Y (normalized to chromosome 20) was used with the *F* stat to define the sample sex as follow: *male*: *F* > 0.6 and normalized Y coverage > 0.1, *female*: *F* < 0.4 and normalized Y coverage < 0.1. Samples with values outside these ranges were labelled as sex unspecific (**Supplementary Figure 13**). Samples were marked as failing hard filters if: a) chromosomal sex was unspecific, b) exhibited sample-specific low call rates (< 0.85) and c) mean coverage on chromosome 20 was equal to zero. **Supplementary Table 1** summarises the number of samples affected per hard filter.

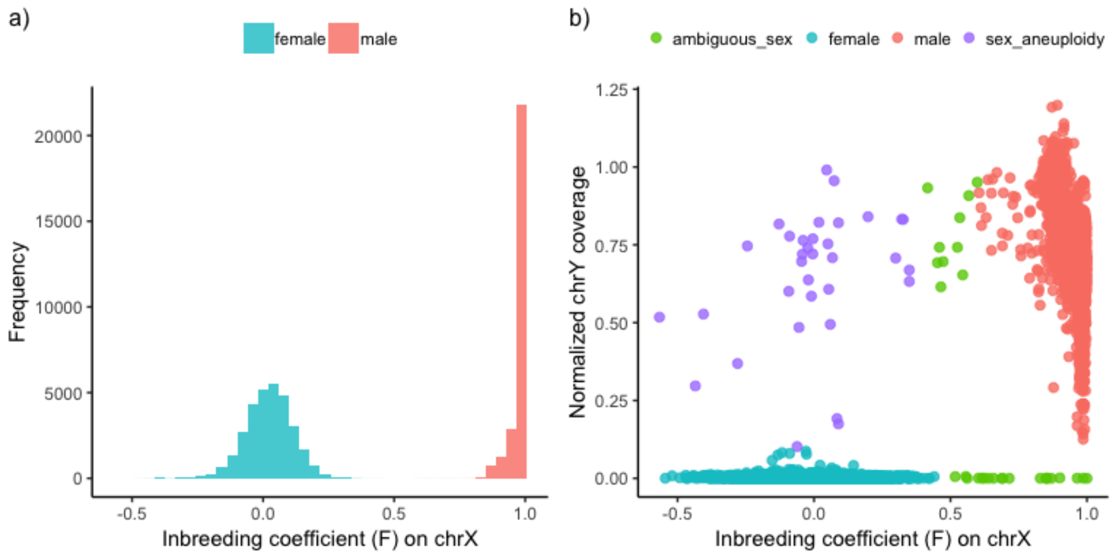

Supplementary Figure 12. a) Inbreeding coefficient (F-stat) distribution computed over 57,628 samples. b) Inbreeding coefficient (x-axis) vs. normalized chromosome Y coverage (y-axis). Sample chromosomal sex was defined as follow, i) female: F < 0.4 and coverage chrY < 0.1, ii) male: F > 0.6 and coverage chrY > 0.1, iii) aneuploidy: F < 0.4 and coverage chrY >= 0.1, iv) samples failing any of these criteria were flagged as 'ambiguous sex'.

Supplementary Table 1. The number of affected samples per hard filter.

Hard filters	N. of samples	Percent (%)
Low call rate	9	0.02
Low coverage	1	0.00
Ambiguous sex	30	0.05
Sex aneuploidy	34	0.06
Filters combined	72	0.12

Inferring population ancestry.

The 1000 Genomes Phase 3 sequence data aligned to the human genome build GRCh38 (European Variation Archive (EVA) accession: PRJEB30460) was used to impute the global ancestry within the samples in the exome sequencing cohort. Both datasets were first merged based on locus and reference/alternate alleles. After merging, the Hail function *hl.hwe_normalized_pca* was used to compute the top 15 principal components on the subset of the well-behaved variants, defined as described above (see Hard filters section). A total of ~76,000 variants were included in the final set.

The set of 2,548 samples with known ancestry (from the 1000 Genomes Phase 3 dataset) was leveraged to build a random forest-based classifier using the top 15 computed principal components (PCs) as input features. Two-thirds of these samples were used as a *training dataset* and the remainder used as a *test dataset*. This step

was combined with a recursive feature (a.k.a principal components) elimination procedure to define the optimal combination of PCs achieving the highest accuracy in the classification on the test data. In addition, a 10-fold cross-validation step was used for tuning the model parameters as previously described[2].

The model achieving the highest accuracy (>0.97) was then used to predict the ancestry of the remaining samples (discovery dataset with unknown ancestry). Each sample was broadly assigned to one of European (EUR), American (AMR), African (AFR), East Asian (EAS) or South Asian (SAS) population labels if random forest probability (p) > 0.8. Samples failing this threshold were labelled as OTHER. **Supplementary Figure 14** and **Supplementary Table 2** summarise the ancestry inference process results. The implemented approach showed high accuracy in classifying samples with reported ethnicity from the UK Biobank cohort (**Supplementary Table 3**).

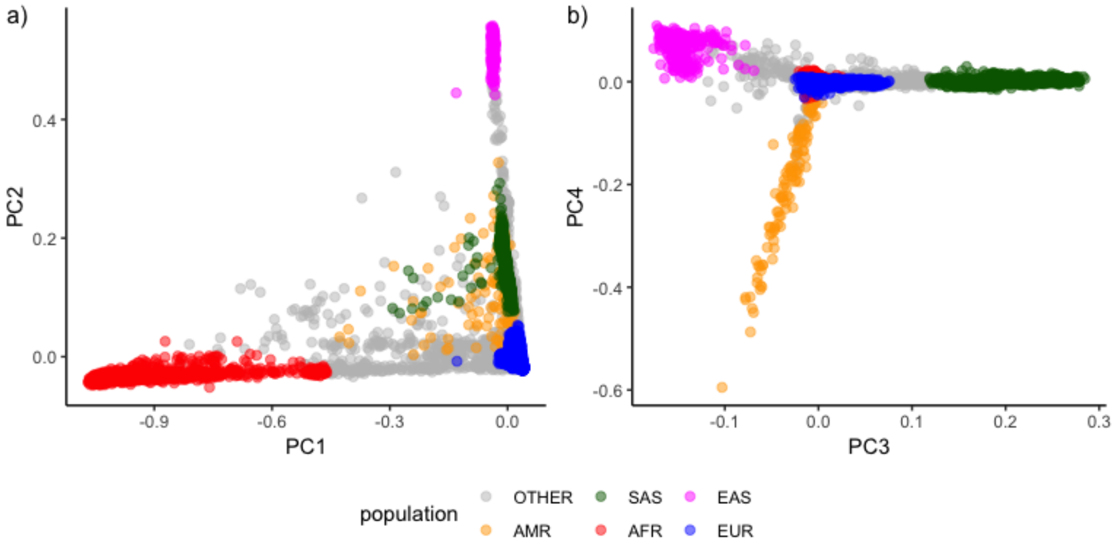

Supplementary Figure 13. Samples projected onto the top four ancestry principal components (PCs) and their classification into five major ancestral populations. Samples were assigned to SAS, EAS, AMR, AFR or EUR if random forest probability (p) > 0.8. Samples failing this threshold were labelled as OTHER (grey). a) PC1 vs PC2 and b) PC3 vs PC4.

Supplementary Table 2. The number of samples assigned per population. As expected, most samples were assigned to European ancestries (~91%). Approximately 3% of the samples were not assigned to a specific population (labelled as OTHER).

Population	N. of samples	Percent (%)
AFR	1,196	2.07
AMR	111	0.19
EAS	313	0.54
EUR	52,844	91.63
OTHER	1,772	3.07
SAS	1,437	2.49

Supplementary Table 3. Confusion matrix with assigned population vs reported ethnicity for samples from the UK Biobank (UKBB).

Assigned population	Reported ethnicity	N. of samples per assigned population	N. of samples per reported ethnicity	Percent true classified (%)
AFR	African	319	332	96.08
EUR	British	42450	43184	98.30
EAS	Chinese	169	173	97.69
SAS	Indian	690	708	97.46
EUR	Irish	1495	1498	99.80
SAS	Pakistani	138	138	100.00

Inferring sample relatedness.

The *hl.pc_relate* function from Hail was used to compute the relatedness between samples. Relatedness was computed among samples passing the hard filters. A variant was considered for inferring relatedness if it met the following criteria: 1) protein-coding exonic variant, 2) autosomal, 3) bi-allelic single nucleotide variants (SNVs), 4) call rate across samples > 95%, 5) allele frequency (internal) > 1% and 6) LD-pruned with a cut-off at *r2* = 0.1. After running *hl.pc_relate*, Hail's *hl.maximal_independent_set* function was used to select the largest set of samples with no pair of samples related at the second-degree relatedness or closer (kinship coefficient > 0.125), prioritising cases over controls. This process filtered out a total of 3,782 samples (either twin/duplicated or first-degree relatives).

Platform inference.

Detailed capture platform meta-data information was missing for a fraction of the samples within the assembled cohort (~20%). To impute a platform for these samples, we adopted the data-driven approach proposed by gnomAD[1]. In brief, a list of the known exome capture intervals across multiple exome capture products was compiled for imputing samples platforms (including Agilent Sure Select All Exons products (version 2 to 5) and IDT xGEN). Only bi-allelic variants falling within these regions were included in the analysis. A sample per interval call-rate matrix was computed by considering the set of biallelic variants within each interval. The call-rate values were further discretised as non-called (0) and called (1) by applying a call-rate cut-off at 0.25 and principal component analysis performed on the discrete matrix. The top seven principal components (variance explained higher than 98%) were used as input for HDBSCAN (https://hdbscan.readthedocs.io), an unsupervised clustering method that allowed us to group and assign generic sample platform labels. **Supplementary Figure 15** shows the samples projected onto principal components two and three. This method assigned the platform accurately for 100% of the samples in the UK Biobank (those with known platform labels), demonstrating the validity of this approach.

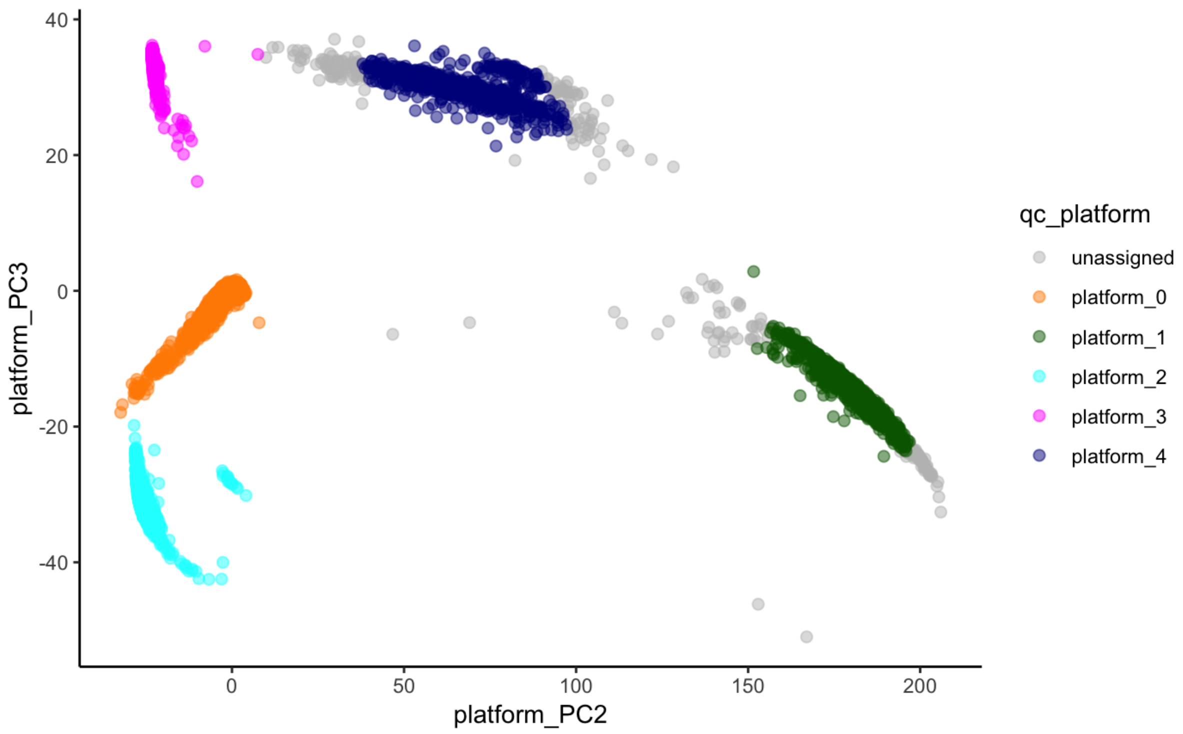

Supplementary Figure 14. Samples projected onto the platform's principal components (PC) two and three. No generic platform (grey dots) was assigned for less than 0.5% of the samples (n=233). The proposed clustering approach accurately assigned 100% of the samples in the UK Biobank cohort (samples with known capture platform information, orange cluster). The exome capture platform intervals used in the analysis are described in the Methods section.

Platform- and population-specific outliers filtering.

Sample ancestry and capture platform are two of the most frequent confounders when analysing exome sequencing data. Thus, we computed a set of sample quality control metrics stratified by population and platform to detect sample outliers. Specifically, we computed the number of deletions, the number of insertions, the number of SNVs, the ratio of deletions to insertions, the ratio of transitions to transversions, and the ratio of heterozygous to homozygous variants using the Hail function *hl.sample_qc*. A sample was marked as an outlier and filtered out if the value for a given QC metric was four median absolute deviations (MAD) from its median. **Supplementary Table 4** summarises the number of samples detected as outliers per evaluated QC metric.

Supplementary Table 4. The number of samples detected as outliers by evaluating different sample quality control (QC) metrics. Samples were grouped as per assigned population/platform, and QC metrics were computed per group. Multiple samples (n=104) were detected as outliers by two or more QC metrics.

QC metrics	N. of sample outliers	Percent (%)
Number of SNPs	134	0.23
Number of deletions	85	0.15
Number of insertions	85	0.15
Ratio transmission/transversion	89	0.15
Ratio insertion/deletion	14	0.02
Ratio heterozygous/homozygous	266	0.46

Final sample QC and evaluation.

After applying the above sample QC steps and filtering out the samples without approval for analysis, our cohort consisted of 49,308 samples (**Supplementary Table 5**). At this stage, multi-allelic variants were split using the Hail function *hl.split_multi_hts,* and the dataset was filtered to high-quality genotypes. Genotypes were defined as high-quality if: a) depth of coverage >= 10, b) genotype quality >= 20 and c) genotype allele balance of heterozygotes > 0.20.

In addition, we evaluated the per sample distribution of the depth of coverage (DP) and genotype quality (GQ) stratified by case/control and male/female status. Our analysis revealed a comparable distribution of these metrics between cases/controls (**Supplementary Figure 16**) and male/females (**Supplementary Figure 17**). Mean DP values ranged between 20-35X (recommended cut-off is >10X) whereas GQ values ranged between 50-80 (recommended cut-off is >20).

Supplementary Table 5. Number of remaining samples after each filter stage. *Population filter refers here to samples with assigned European ancestries.

Filter stages	Remaining samples
Unfiltered	57,628
Hard filters	57,560
Hard filters, relatedness	53,862
Hard filters, relatedness, QC outliers	53,507
Hard filters, relatedness, QC outliers, *population	49,308

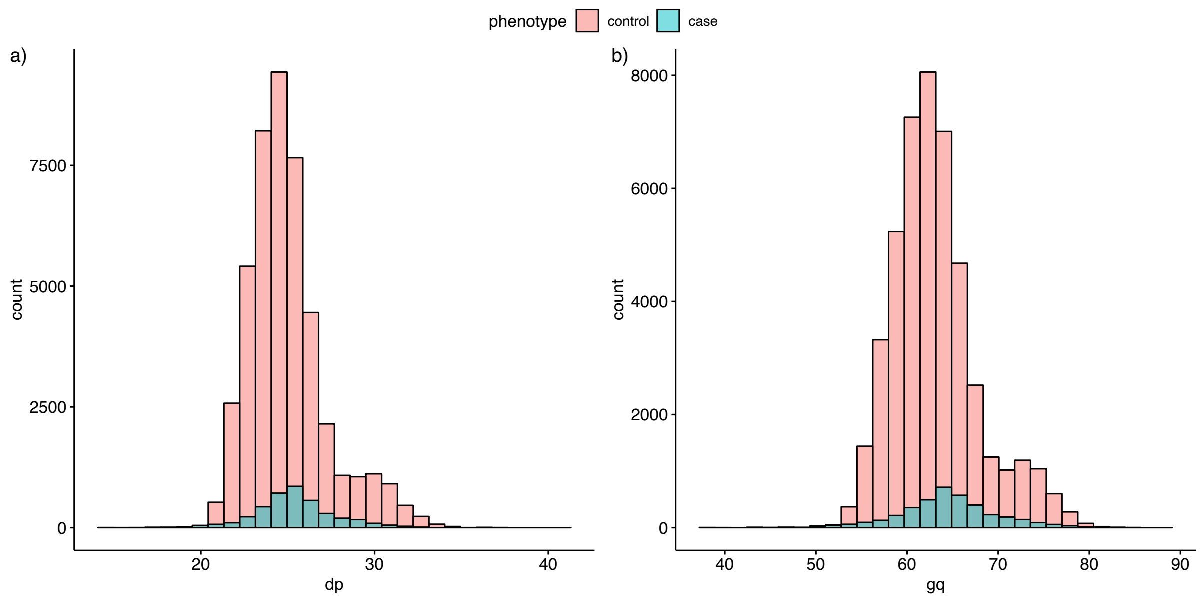

Supplementary Figure 15. Distribution of per sample averaged QC metrics stratified by phenotype (case/control). a) Mean depth of coverage (DP) and b) Mean genotype quality (GQ). QC metrics were computed per sample across autosomal variants.

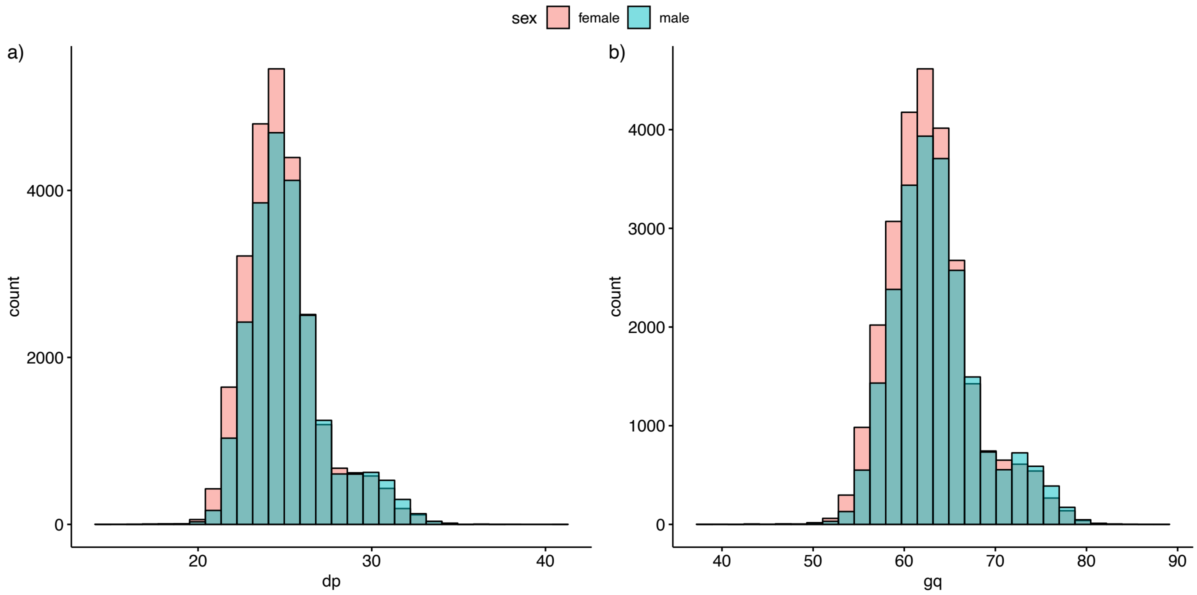

Supplementary Figure 16. Distribution of per sample averaged QC metrics stratified by sex (female/male). a) Mean depth of coverage (DP) and b) Mean genotype quality (GQ). QC metrics were computed per sample across autosomal variants.

Variant QC

To define a set of high-quality variants for downstream analysis, we then applied several QC steps to the variants present in samples passing the sample QC process.

Hard filters.

We followed the variant QC scheme proposed by Karczewski *et al.*[1], where variants were flagged as failing hard filters if they showed a) an excess of heterozygotes (inbreeding coefficient < -0.3) and b) an absence of at least one sample with a high-quality genotype (allele-count zero, as defined above).

RF model.

A random forest (RF) model was trained and applied to distinguish true variations from potential false positives[1]. Positive training sets were downloaded from gnomAD repository (gs://gcp-public-data--gnomad/truth-sets/hail-0.2). Variants failing traditional GATK hard filters (QD < 2 or FS > 60 or MQ < 30) were used as a negative training set. Allele- and site-specific sequencing quality metrics were used as features for training the model (**Supplementary Table 6**). Features were imputed using its median where the value was missing. The chromosome 20 (test set) was left out of the training process for evaluation proposes. The final RF model achieved an accuracy >0.97 on this set of variants (test set). A variant was filtered out if the RF probability of being false positive was higher than 0.8.

Supplementary Table 6. Features used in the random forest model to predict the variant probability of being true positive or false positive.

RF features	Description	Importance
variant_type	SNV or indel	0.011
SOR	Symmetric Odds Ratio of 2x2 contingency table to detect strand bias	0.105
ReadPosRankSum	Z-score from Wilcoxon rank-sum test of Alt vs Ref read position bias	0.016
InbreedingCoeff	Inbreeding coefficient as estimated from the genotype likelihoods per-sample when compared against the Hardy-Weinberg expectation	0.103
FS	Phred-scaled p-value using Fisher's exact test to detect strand bias	0.041
DP	Approximate read depth	0.003
QD	Allele-specific Variant Confidence/Quality by Depth	0.704

was_mixed	True if both SNVs and indels are present at the site	0.001
n_alt_alleles	Number of alleles at the site	0.001
MQRankSum	Z-score From Wilcoxon rank-sum test of Alt vs Ref read mapping qualities	0.010

VQSR filter.

In addition to the proposed RF model, we applied the conventional GATK Variant Quality Score Recalibration (VQSR) as a complementary approach to filter out low-quality variants. We used the recommended annotations and training datasets as suggested by the GATK best practices (https://gatkforums.broadinstitute.org/gatk). Both SNVs and indels were excluded if they failed the VQSR filter, according to the default settings. This allowed us to identify a fraction of variants that were likely false positives that passed the RF filter (**Supplementary Figure 18**).

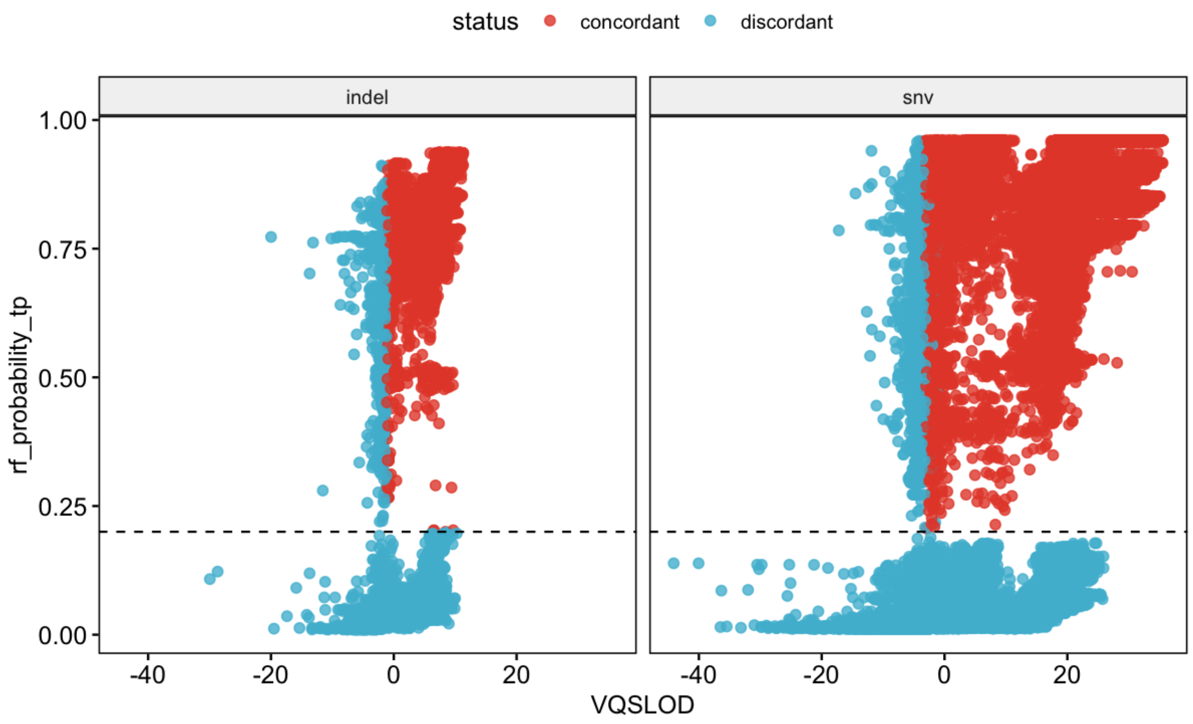

Supplementary Figure 17. Variant quality score recalibration (x-axis) vs Random Forest (RF) probability of being true positive (y-axis). Variants (SNVs and indels) are depicted for chromosome 20. The dashed line indicates the cut-off used for the RF probability (=0.2). Concordant (red dots): variants pass both the RF and VQSR filters; discordant (blue dots): variants fail at least one of the RF or VQSR filters.

Coverage.

Finally, we defined a variant as passing the QC if a) was covered by the major capture platforms used in the assembled cohort (different versions of Agilent Sure Select All Exome and IDT xGen panel 1 and b) showed coverage of 10X or more in at least the 90% of the samples in the gnomAD genome (version 3.1.0) and the current cohort. **Supplementary Figure 19** shows coverage information between cases and controls for known and candidate CHD genes.

Supplementary Table 7 summarises the number of variants affected by each applied filter and the final number of variants considered for further analysis.

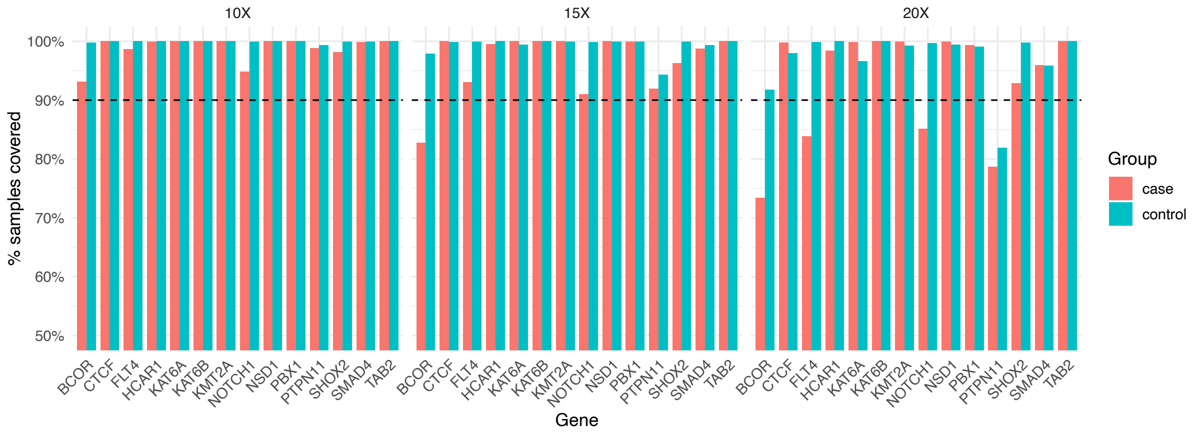

Supplementary Figure 18. Coverage harmonization between cases and controls for burden-tested genes. Bars show the average percentage of samples achieving sequencing depth ≥10X, ≥15X, and ≥20X across variants included in the burden analysis, stratified by cases (blue) and controls (orange). At the 10× threshold, coverage exceeded 90% for both cases and controls across all genes, with minimal case-control differences. At higher thresholds (15X and 20X), coverage decreased for several genes, but case and control groups remained closely matched, indicating that relative harmonization was preserved and technical depth differences are unlikely to bias burden test results.

Supplementary Table 7. The number of remaining variants per filter stage. RF: Random Forest filter, VQSR: Variant Quality Score Recalibration, Coverage: >10X in at least 90% of the samples in gnomAD genome cohort.

**Filter stages**	**Remaining variants**
Unfiltered	11,433,645
Hard filters	11,406,658
Hard filters, RF	9,490,151
Hard filters, RF, VQSR	9,191,448
Hard filters, RF, VQSR, Coverage	9,134,464

Variant annotation

The cohort-VCF file was annotated using the Variant Effect Predictor tool (API version 95) with the flag *--everything*. The most severe variant consequence per protein-coding transcript was considered. The variant consequence severity was set based on the severity rank from Ensembl (https://www.ensembl.org), which prioritise variants as follows: protein-truncating > protein-altering > synonymous variants. The VEP tool functionalities were extended by using the plug-ins CADD (version 1.6) and dbNSFP[3] (version 4.1a) to annotate different missense variant pathogenicity scores (CADD[4], MPC[5], REVEL[6] and MVP[7]).

Calibration of gene-based burden tests

To assess whether the observed genomic inflation in gene-based burden tests is attributable to the case-control imbalance rather than population stratification or technical confounding, we performed two complementary calibration analyses: MAF-stratified evaluation of synonymous variant test statistics and label-permutation null tests.

Supplementary Table 8. MAF-stratified genomic inflation metrics for synonymous variant burden tests. Gene-level Fisher's exact test statistics (lambda_GC and lambda_1000) for synonymous variants, stratified by minor allele frequency bins using cohort-internal allele frequency (top) and gnomAD genomes allele frequency (bottom). Results are shown for all CHD (aCHD), non-syndromic CHD (nsCHD), and syndromic CHD (sCHD) versus controls. N genes indicates the number of genes with at least one qualifying variant per bin.

Internal AF stratification:

Comparison	MAF bin	N genes	lambda_GC	lambda_1000
aCHD	<= 5e-05	12,837	2.04	1.07
aCHD	(5e-05, 1e-04]	6,225	1.29	1.02

aCHD	(1e-04, 1e-03]	6,396	1.22	1.02
aCHD	(1e-03, 1e-02]	96	4.61	1.25
nsCHD	<= 5e-05	11,770	2.48	1.16
nsCHD	(5e-05, 1e-04]	4,423	1.52	1.06
nsCHD	(1e-04, 1e-03]	4,403	1.34	1.04
nsCHD	(1e-03, 1e-02]	80	4.22	1.35
sCHD	<= 5e-05	9,135	1.74	1.13
sCHD	(5e-05, 1e-04]	4,720	1.99	1.17
sCHD	(1e-04, 1e-03]	4,871	1.32	1.06
sCHD	(1e-03, 1e-02]	79	0.76	0.96

gnomAD genomes AF stratification:

Comparison	MAF bin	N genes	lambda_GC	lambda_1000
aCHD	<= 5e-05	11,042	1.28	1.02
aCHD	(5e-05, 1e-04]	5,040	1.80	1.06
aCHD	(1e-04, 1e-03]	4,937	2.02	1.07
aCHD	(1e-03, 1e-02]	550	4.51	1.25
nsCHD	<= 5e-05	9,311	1.39	1.04
nsCHD	(5e-05, 1e-04]	3,705	2.12	1.12
nsCHD	(1e-04, 1e-03]	3,738	2.51	1.16
nsCHD	(1e-03, 1e-02]	383	5.99	1.55
sCHD	<= 5e-05	7,783	1.52	1.09
sCHD	(5e-05, 1e-04]	3,415	2.18	1.21
sCHD	(1e-04, 1e-03]	3,152	2.18	1.21
sCHD	(1e-03, 1e-02]	247	5.63	1.81

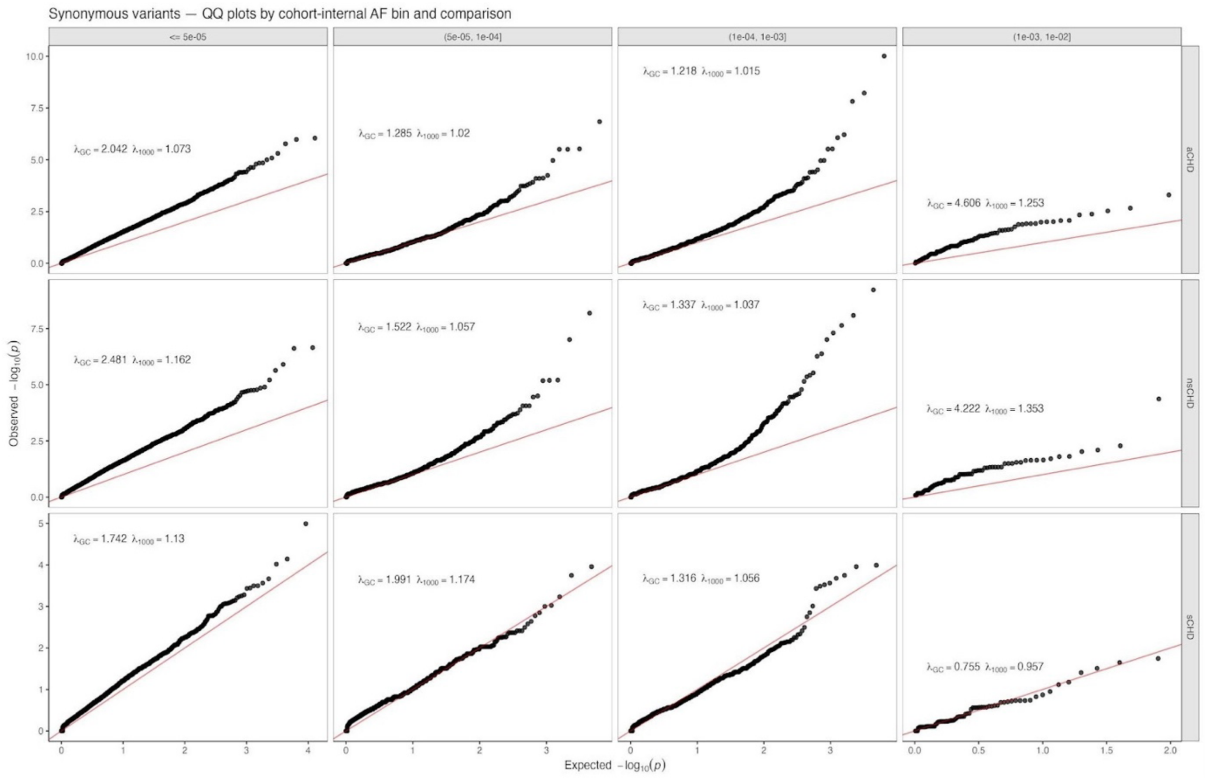

Supplementary Figure 19. MAF-stratified QQ plots of synonymous variant burden tests using cohort-internal allele frequency. Quantile-quantile plots of gene-level Fisher's exact test p-values for synonymous variants, stratified by cohort-internal allele frequency into four bins. Rows correspond to the three case-control comparisons: all CHD, non-syndromic CHD, and syndromic CHD versus controls. The red diagonal line indicates the expected uniform distribution under the null hypothesis. Lambda_GC and lambda_1000 are shown per panel.

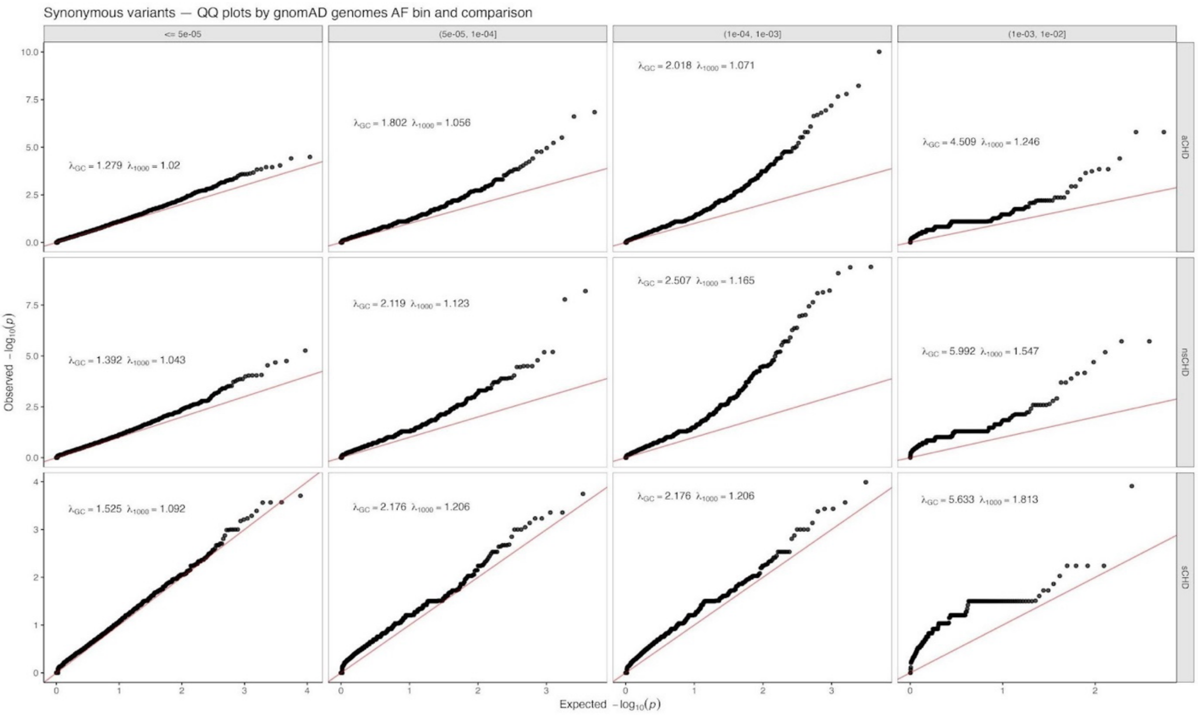

Supplementary Figure 20. MAF-stratified QQ plots of synonymous variant burden tests using gnomAD genomes allele frequency. Quantile-quantile plots of gene-level Fisher's exact test p-values for synonymous variants, stratified by cohort-internal allele frequency into four bins. Rows correspond to the three case-control comparisons: all CHD, non-syndromic CHD, and syndromic CHD versus controls. The red diagonal line indicates the expected uniform distribution under the null hypothesis. Lambda_GC and lambda_1000 are shown per panel.

Supplementary Table 9. Label-permutation null test for genomic inflation. Observed lambda_GC and lambda_1000 values compared with null distributions derived from 1,000 random permutations of case-control labels. Null mean and standard deviation (SD) are reported for each metric. Empirical p-values indicate whether observed inflation exceeds the permutation null.

Comparison	Observed lambda_GC	Observed lambda_1000	Null mean (SD) lambda_GC	Null mean (SD) lambda_1000	Empirical p
aCHD	1.35	1.04	0.97 (0.02)	0.998 (0.002)	< 0.001
nsCHD	1.47	1.05	0.98 (0.03)	0.998 (0.003)	< 0.001
sCHD	1.35	1.04	1.01 (0.02)	1.002 (0.004)	< 0.001

References

1. Karczewski, K. J. *et al.* The mutational constraint spectrum quantified from variation in 141,456 humans. *Nature* **581**, 434–443 (2020).
2. Perez-Riverol, Y., Kuhn, M., Vizcaíno, J. A., Hitz, M. P. & Audain, E. Accurate and fast feature selection workflow for high-dimensional omics data. *PLoS One* **12**, e0189875 (2017).
3. Liu, X., Li, C., Mou, C., Dong, Y. & Tu, Y. dbNSFP v4: a comprehensive database of transcript-specific functional predictions and annotations for human non-synonymous and splice-site SNVs. *Genome Med.* **12**, 103 (2020).
4. Rentzsch, P., Witten, D., Cooper, G. M., Shendure, J. & Kircher, M. CADD: predicting the deleteriousness of variants throughout the human genome. *Nucleic Acids Res.* **47**, D886–D894 (2019).
5. Samocha, K. E. *et al.* Regional missense constraint improves variant deleteriousness prediction. *bioRxiv* 148353 (2017). doi:10.1101/148353
6. Ioannidis, N. M. *et al.* REVEL: An Ensemble Method for Predicting the Pathogenicity of Rare Missense Variants. *Am. J. Hum. Genet.* **99**, 877–885 (2016).
7. Qi, H. *et al.* MVP predicts the pathogenicity of missense variants by deep learning. *Nat. Commun.* **12**, 510 (2021).
